# Five-Year Relative Survival Rates of Women Diagnosed with Uterine Cancer by County-Level Socioeconomic Status Overall and across Histology and Race/Ethnicity

**DOI:** 10.3390/cancers16152747

**Published:** 2024-08-01

**Authors:** Akemi T. Wijayabahu, Jennifer K. McGee-Avila, Meredith S. Shiels, Alfonsus Adrian H. Harsono, Rebecca C. Arend, Megan A. Clarke

**Affiliations:** 1 Division of Cancer Epidemiology and Genetics, National Cancer Institute, Rockville, MD 20850, USA; jennifer.mcgee-avila@nih.gov (J.K.M.-A.); shielsms@mail.nih.gov (M.S.S.); megan.clarke@nih.gov (M.A.C.); 2 Division of Gynecologic Oncology, Department of Obstetrics and Gynecology, University of Alabama at Birmingham, Birmingham, AL 35233, USA; aharsono@uabmc.edu (A.A.H.H.); rarend@uabmc.edu (R.C.A.)

**Keywords:** socioeconomic status, uterine cancer, race/ethnicity, histologic subtype, five-year survival, race/ethnic disparity

## Abstract

**Simple Summary:**

Persistent survival disparities by racial/ethnic groups are expected to worsen with rising incidence rates of aggressive uterine cancer subtypes. Understanding the impact of area-based socioeconomic factors on survival outcomes may help to better understand these disparities. This research aims to understand how living in counties with lower educational attainment, higher poverty, higher unemployment, lower median household income, and population density in urban areas may impact survival rates of women diagnosed with uterine cancer across racial/ethnic groups. Our findings show that lower county-level socioeconomic characteristics are linked with worse survival rates, mainly impacting women diagnosed with aggressive histologic subtypes and underrepresented racial/ethnic groups. Regardless of tumor and socioeconomic characteristics, non-Hispanic (NH) Black women consistently experience the poorest survival outcomes compared to other racial/ethnic groups included in this study. Racial/ethnic disparities in survival were observed even in the most affluent counties, suggesting that other factors beyond county-level socioeconomic status are at play.

**Abstract:**

Understanding socioeconomic factors contributing to uterine cancer survival disparities is crucial, especially given the increasing incidence of uterine cancer, which disproportionately impacts racial/ethnic groups. We investigated the impact of county-level socioeconomic factors on five-year survival rates of uterine cancer overall and by histology across race/ethnicity. We included 333,013 women aged ≥ 30 years with microscopically confirmed uterine cancers (2000–2018) from the Surveillance, Epidemiology, and End Results 22 database followed through 2019. Age-standardized five-year relative survival rates were compared within race/ethnicity and histology, examining the differences across tertiles of county-level percent (%) <high-school education, %<150 percent poverty, %unemployment, median household income, and %urbanicity. Overall age-adjusted five-year relative survival was 77.7%. Rates were lowest among those residing in the least advantaged counties (tertile 3) and highest among the most advantaged (tertile 1): education (74.7% vs. 80.2%), poverty (72.9% vs. 79.8%), unemployment (75.7% vs. 80.5%), and income (73.3% tertile 1 vs. 78.1% tertile 3). Impact of county-level socioeconomic characteristics on survival across histology was minimal. We observed considerable survival disparities among NH-Black and NH-Native American/Alaskan Native women, regardless of tumor and socioeconomic characteristics. These findings add to our understanding of how county-level socioeconomic characteristics affect uterine cancer survival inequalities among racial/ethnic groups.

## 1. Introduction

Rising incidence and mortality rates of uterine cancer pose a significant public health challenge in the United States (U.S.), with an estimated 67,880 cases and 13,250 deaths projected for 2024 [1]. While the overall five-year relative survival rate for uterine cancer is favorable at 83.3%, survival varies significantly by stage at diagnosis (according to Summary Stage, localized: 95.4%, regional: 69.9%, distant: 18.1%) and histologic subtype (endometrioid: 91.8%, non-endometrioid: 57.5%, sarcoma: 52.8%) [2]. Further, while for most cancers five-year relative survival rates have improved over time, uterine cancer survival has decreased [1], likely owing to an increasing incidence of non-endometrioid subtypes within the last two decades, which have poorer prognosis and are more common in non-Hispanic (NH) Black women. Studies dating back to 1987 consistently highlighted racial differences in survival outcomes among Black women with uterine cancer compared to White women, particularly among women who did not receive recommended treatment [3]. More recent studies have shown that survival differences across racial/ethnic groups persist even after controlling for tumor characteristics, time to treatment, and type of treatment [4,5,6]. Therefore, it is imperative to investigate and understand other factors associated with these survival disparities, particularly in light of the increasing incidence rates of aggressive histologic subtypes [2,7].

Lower socioeconomic status, whether defined individually or as a group (e.g., based on the lived-in area, including county-level socioeconomic status as an indication of socioeconomic deprivation), is typically marked by lower educational attainment, higher unemployment, and lower income levels [8]. Living in areas with socioeconomic deprivation has been linked with poor survival outcomes in women with uterine cancer [8,9,10]. Lower area-based socioeconomic status has also been associated with delayed healthcare utilization and access, resulting in advanced stage at uterine cancer diagnosis [11]. Black and Hispanic women are more likely to present with advanced disease compared to White women [12]. Socioeconomic deprivation, often manifested through residential segregation, is linked to the differential distribution in lifestyle factors (e.g., adiposity, parity) [13,14], chronic comorbidities [15], and other potential risk exposures (e.g., air pollutants) [16,17,18] among racial/ethnic groups. Moreover, inequality in access to healthcare further exacerbates these disparities, and may contribute to the observed differences in survival across racial and ethnic groups [19,20].

To our knowledge, prior studies have not compared uterine cancer survival rates across histologic subtypes, socioeconomic factors, and racial/ethnic groups using a large, population-based database. It is important to investigate the intersectionality of these factors to gain a deeper understanding of healthcare outcome disparities. Thus, our objective was to compute age-standardized five-year relative survival rates for women diagnosed with uterine cancer, by county-level socioeconomic factors (education, poverty, unemployment, income, urbanization) overall, and by histologic subtype across racial/ethnic groups. 

## 2. Materials and Methods

### 2.1. Data Source 

We utilized the Surveillance, Epidemiology, and End Results (SEER) Research Plus Limited-Field Database 22 (SEER 22), excluding Illinois and Massachusetts, which aggregates incident uterine cancer cases from 20 population-based cancer registries across the United States and linked with the American Census Survey (ACS) database [21]. These registries collectively cover approximately 41.9% of the total U.S. population based on the 2020 U.S. Census [21]. All data utilized in this study are de-identified and are publicly available; thus, the study was exempt from institutional review board approval and the need for informed consent. 

### 2.2. Case Selection and Study Design

**Figure 1:**
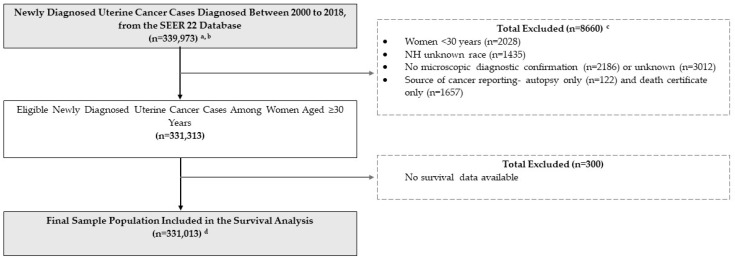
Flow chart of study population selection: women diagnosed with uterine cancer among U.S. women aged ≥ 30 years reported in the SEER 22 database. Abbreviations: SEER (Surveillance, Epidemiology, and End Results). Notes: ^a^ We utilized the Surveillance, Epidemiology, and End Results (SEER 22) database, excluding Illinois and Massachusetts, which combines 20 population-based cancer registries and links to American Community Survey data. ^b^ All cases included are first matching primary record of malignant uterine cancers (corpus uteri and uteri, not otherwise specified). ^c^ Some cases have one or more overlapping exclusion criteria. Thus, the total number excluded is less than the sum of individual categories of exclusion. ^d^ Final sample included in our survival analysis. Survival calculated cases diagnosed between 2000 and 2018, and survival data available from 2000 through 2019.

In this retrospective cohort study of uterine cancer cases from the SEER database, we included malignant corpus uteri and uterine corpus, NOS (International Classification of Diseases for Oncology Site, Third Edition (ICD-O-3) codes: C540–C549, and C559) diagnosed between 2000 and 2018 in women aged 30 years and older. The rationale for the age restriction is to balance between minimizing the contribution of hereditary cancers and capturing a wider age range comparable to previous studies [2,22]. Cases were restricted to microscopically confirmed first primary uterine cancers. We included racial/ethnic categories provided in SEER, including NH-White, NH-Black, NH-American Indian or Alaska Native (hereafter referred to as Native American/Alaska Native, NA/AN), and Hispanic women, excluding unknown races (n = 1435). We also excluded cases identified with autopsy only or death certificates only, and those with no survival data. Case selection is depicted in the flow chart shown in Figure 1.

### 2.3. Demographic and Clinical Characteristics

We included race and ethnicity categories from the SEER database [23] (NH-White, NH-Black, NH-Asian/PI, NH-NA/AN, and Hispanic women). Race and ethnicity data in cancer registries are obtained from patient medical records, which are primarily based on self-reported data, caretaker reports, and physical appearance [24]. We grouped histologic subtypes as endometrioid, non-endometrioid, sarcomas, and other cancers, as shown in Table S1. We included stage at diagnosis (localized, which corresponds to FIGO Stage I, regional to FIGO Stage II–III, and distant to FIGO Stage IV) using SEER summary stage from the diagnosis period 2004 to 2018, because stage information was not available prior to 2004 [25,26]. In addition to the n = 54,918 women with cancers diagnosed before 2004, we set n = 10,978 cases classified as unknown/not staged, and n = 10 tumors classified as in situ according to the SEER summary stage diagnosed from 2004 to 2018 as missing for analyses evaluating rates by stage only. 

### 2.4. County-Level Socioeconomic Characteristics 

County-level socioeconomic characteristics including percent with less than a high-school education (<HS education), percentage of persons below 150 percent poverty line (<150% poverty), percentage of unemployment (unemployment), and median household income in U.S. dollars (USD) were selected from the 2015–2019 ACS (American Community Survey, conducted by the U. S. Census Bureau) cycle, linked with the SEER 22 database. We selected 2015–2019 ACS cycle data, the most recent county-level socioeconomic estimates that overlaps with our study population. The socioeconomic characteristics from the 2015–2019 ACS cycle are highly correlated with those from the 2000s, 2007–2011, and 2010–2014 ACS cycles. We chose these county-level socioeconomic characteristics because these area-based socioeconomic indicators effectively detect health outcome differences and monitor disparities within healthcare systems [27]. For each variable, we ranked the counties from lowest to highest values, and created tertiles based on this ranked distribution, with cutoff values identified for each tertile [28]. Tertile cutoff values for percentage of people with <HS education are <9.48% (Tertile 1), 9.48–14.74% (Tertile 2), and >14.74% (Tertile 3); percentage of people < 150% poverty level < 20.92% (Tertile 1), 20.92–28.12% (Tertile 2) and >28.12% (Tertile 3); percent unemployment < 4.08% (Tertile 1), 4.08–5.86% (Tertile 2), >5.86% (T3); and median household income < USD 47,060 (Tertile 3), USD 47,060–USD 56,590 (Tertile 2), >USD 56,590 (Tertile 1). Tertile distributions correspond to the county-level socioeconomic measure in the U.S. population and not the distribution of women with uterine cancer included in our study population. For any given socioeconomic measure, except for the median household income variable, tertile 1 corresponds to the first 33% of counties with the highest socioeconomic status, while tertiles 2 and 3 correspond to medium and the lowest socioeconomic status. Median household income is inverse, where tertile 3 corresponds to the highest income. 

Additionally, we included the %urban (=total urban population/total population) measure from the 2010 U.S. Census, linked with the SEER 22 database, which denotes the percentage of individuals residing in urban areas within each county. We categorized the %urban measure into tertiles based on the distribution of counties as follows: <24.53%, 24.53–57.02%, and >57.02%.

### 2.5. Statistical Analysis 

Five-year relative survival rates with a 95% CI were calculated using SEER*Stat survival session. Expected survival rates considered age-specific all-cause mortality rates within the U.S. reference population, utilizing actuarial and Ederer II methods. These computations account for survival variations due to racial/ethnic composition, geographic area, and socioeconomic status [29]. We included women diagnosed with uterine cancer between 2000 and 2018 and assessed survival from 2000 to 2019. To facilitate comparisons between participant characteristics, we displayed age-standardized five-year survival estimates in the main tables and figures, and include non-age standardized relative survival estimates in the Supplementary Materials. 

We computed age-standardized five-year relative survival estimates for socioeconomic characteristics for the overall study population, and we performed stratified analyses by histology and race/ethnicity. We did not report survival rates or specific case/death counts for groups with fewer than 16 cases and/or 16 deaths overall [30]. Due to the small sample size across multiple strata, we did not include in the figures the survival rates for the %urban measure across racial/ethnic groups, nor the survival rates for non-endometrioid histology among NH-NA/AN women. Additionally, we restricted our stratified analysis by histology to the two most common histologic subtypes (endometrioid and non-endometrioid) due to the small sample sizes within stratum-specific categories for rare sarcomas and other heterogeneous histologic types.

Statistically significant group differences in the five-year relative survival estimates were determined using *p*-values calculated from the z-test generated by the SEER*Stat program. We calculated survival rate ratios (SRRs) to assess the magnitude of impact of the different levels of socioeconomic characteristics with respect to the reference group on five-year relative survival estimates. All analyses were conducted using the SEER*Stat software version 8.4.3. All statistical tests were two sided, and statistical significance was assessed at an α level of *p* < 0.05.

Post hoc analyses: We conducted two post hoc analyses. In our first post hoc analysis, we excluded all women with the “other uterine cancer” histologic subtype, which includes heterogeneous cancers (n = 7543, 3%), and other histologic subtypes with possible cancer site misclassification: ICD-O-3 histologic codes 8210/3, 8263/3, 8262/3, 8261/3, 8211/3, 8490/3, and 8141/3 from endometrioid cancer estimates (n = 1530, 0.6%), and 8981/3 from non-endometrioid estimates (n = 16, 0.03%). In our second post hoc analysis, we evaluated the impact of county-level socioeconomic characteristics and survival rates by U.S. geographic regions [31]. We classified SEER registries into three categories, including Northeast (Connecticut, New Jersey, New York), South (Georgia, Kentucky, Louisiana and Texas), Combined Midwest (Iowa), and West (Alaska, California, Hawaii, Idaho, New Mexico, Utah, Washington), and evaluated the five-year relative survival across socioeconomic characteristics.

## 3. Results

### 3.1. Demographic, Clinical, and Socioeconomic Characteristics of the Study Population

Population estimates and five-year relative survival rates are shown in Table 1 and Table S2. Of the 333,013 eligible women diagnosed with uterine cancer between 2000 and 2018, 59% were of ages 50 to 69 years, 69% were NH-White, 75% had endometrioid histology, and 70% presented with localized disease. Counties with mid (Tertile 2) and highest (Tertile 3) proportions of the population with <HS education accounted for 35% to 36% of women with uterine cancer in this study, respectively. Around half of the women with uterine cancer included in this study lived in counties with the lowest percentage of the population living at < 150% poverty line and within mid-level %unemployment. The majority of the study population was from counties with a median household income above USD 56,590 and highest %urban. Most NH-Black, Hispanic, and NH-NA/AN women with uterine cancer resided in counties with lower socioeconomic status compared to other NH-White and NH-Asian/PI women (Figure S1). The majority of women across all racial/ethnic groups lived in counties with higher %urban (Figure S1e).

### 3.2. Overall Five-Year Relative Survival across Demographics and Clinical Characteristics 

The overall age-standardized five-year relative survival rate for women diagnosed with uterine cancer between 2000 and 2018 was 77.7% (95% CI 77.5%, 77.9%) (Table 1, non-age-standardized estimates are shown in Table S2). Overall, age-standardized five-year survival rates were lowest among NH-Black (57.7%) followed by Hispanic (73.1%), NH-Asian/PI (75.8%), NH-NA/AN (77.1%), and NH-White women (81.2%). For the defined histologic subtypes, survival was lowest among women diagnosed with sarcomas (44.4%), followed by non-endometrioid (55.7%) and endometrioid subtypes (87.1%). As expected, survival decreased with more advanced stage (localized 93.0%, regional 64.6%, and distant 16.6%). The observed survival differences based on tumor stage remained consistent across histologic subtypes and racial/ethnic groups (Figure S2). 

### 3.3. Five-Year Relative Survival across Socioeconomic Characteristics among All Women

For all county-level measures of socioeconomic characteristics, survival rates were highest among those residing in the most advantaged counties compared with those living in the least advantaged counties. Except for the %urban category, all other socioeconomic categories followed a dose–response relationship, wherein survival rates declined sequentially with decreasing socioeconomic level (increasing disadvantage). County-level poverty (%<150 percent poverty) showed the greatest impact on survival rates based on absolute differences (79.8% in tertile 1 vs. 72.9% in tertile 3), and a similar impact was observed for %<HS education (80.2% vs. 74.7%), median household income (78.1% vs. 73.3%), and %unemployment (80.5% vs. 75.7%). We did not observe significant differences across tertiles of the %urban measure. The impact of socioeconomic status on survival was consistent across the stage of diagnosis, reflecting the overall patterns (Figure S3). Notably, the poor survival observed among NH-Black women persisted across all stages of diagnosis (Figure S3).

### 3.4. Race/Ethnic Groups Specific Five-Year Relative Survival across County-Level Socioeconomic Characteristics, for All Histologic Subtypes Combined

**Figure 2:**
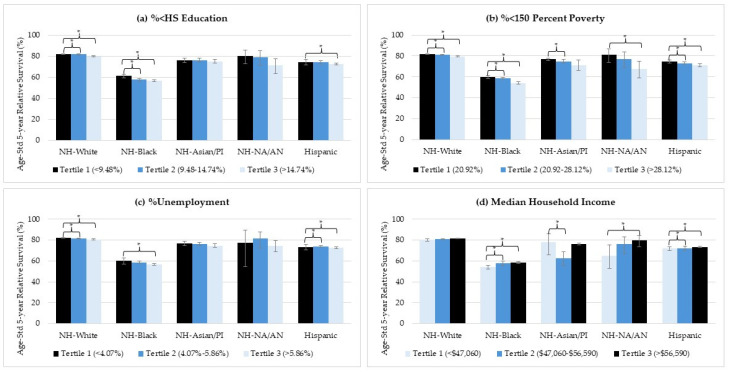
Overall race/ethnicity specific age-standardized 5-year survival rates across socioeconomic characteristics in women diagnosed with uterine cancer between 2000 and 2018, with survival assessed through 2019. Crossbars (*) indicate z-value-based statistical significance (*p* < 0.05), and error bars indicate 95% confidence intervals. Median household income is shown in U.S. dollars (USD denoted as $). Survival estimates for NH-NA/AN (%unemployment tertile 1) is based on small sample sizes (<100 cases and ≤20 deaths).

Age-standardized five-year survival rates declined with increasing county-level socioeconomic disadvantage (lower %<HS education, higher %<150 percent poverty, higher %unemployment, and lower median household income) among almost all racial/ethnic groups (Figure 2, with detailed results in Table S3). Although NH-Black women exhibited the worst survival rates across all socioeconomic characteristics, the impact of county-level socioeconomic characteristics appeared to be largest among NH-NA/AN women with uterine cancer, with the largest absolute differences from most to least advantaged observed for median household income (79.4% vs. 65.0%, *p* = 0.022), %<150 percent poverty (81.4% vs. 67.6%, *p* = 0.026) and %<HS education (80.1% vs. 71.0%, *p* = 0.156; not significant). Among other racial/ethnic groups, %<150 percent poverty and %<HS education were associated with the greatest differences in survival among NH-Black women (59.9% vs. 54.1% and 61.0% vs. 56.7%, respectively; both *p* < 0.001). Although NH-Asian/PI women residing in counties with higher %<150 percent poverty showed a dose–response relationship, the survival difference was significant only in the second tertile compared to the first tertile (76.7% vs. 74.9%, *p* = 0.006), and not the third tertile (76.7% vs. 71.2%, *p* = 0.507), likely due to only 5% of the population being in the third tertile (Figure S1). NH-Asian/PI women residing in counties within the second tertile of median household income had much lower survival (62.6%) compared to those in the first tertile (77.8%) and third tertile (76.1%), with a significant difference observed only between the first and second tertiles (*p* = 0.027). This disparity is likely due to population distribution, with 1% (n = 219) in the first tertile, 3% (n = 542) in the second tertile, and the majority in counties with median household income above USD 56,600 (Figure S1). The absolute differences in survival across county-level socioeconomic characteristics were small, though statistically significant, among NH-White and Hispanic women for nearly all measures (Figure 2). Survival estimates for %urban are not discussed further due to the majority residing in counties with higher %urban (Figure S1) and small sample sizes across multiple strata (Tables S3 and S4).

### 3.5. Five-Year Relative Survival across County-Level Socioeconomic Characteristics Overall and by Histologic Subtype and Race/Ethnicity

**Figure 3:**
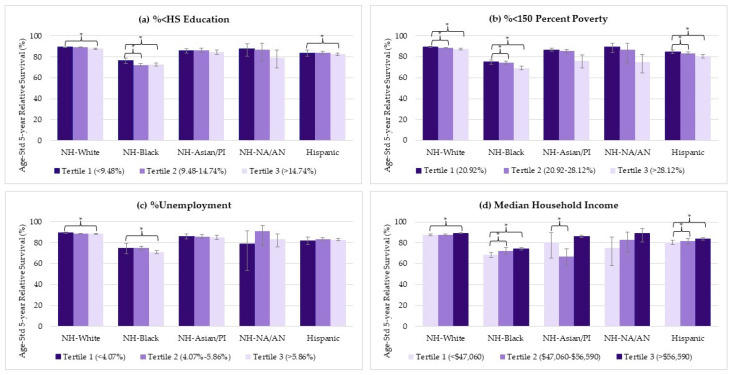
Race/ethnicity specific age-standardized 5-year survival rates across socioeconomic characteristics in women with endometrioid subtype of uterine cancer diagnosed between 2000 and 2018, with survival assessed through 2019. Crossbars (*) indicate z-value-based statistical significance (*p* < 0.05), and error bars indicate 95% confidence intervals. Median household income is shown in U.S. dollars (USD denoted as $). Survival estimates for NH-NA/AN (%unemployment tertile 1) and the NH-Asian/PI (median household income tertiles 1) are based on small sample sizes (<100 cases and/or ≤20 deaths).

**Figure 4:**
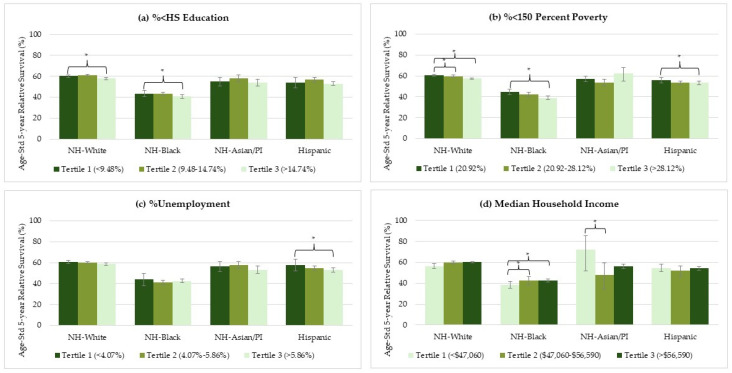
Race/ethnicity specific age-standardized 5-year survival rates across socioeconomic characteristics in women with non-endometrioid subtype of uterine cancer diagnosed between 2000 and 2018, with survival assessed through 2019. Crossbars (*) indicate z-value-based statistical significance (*p* < 0.05), and error bars indicate 95% confidence intervals. Median household income is shown in U.S. dollars (USD denoted as $). Survival estimates for NH-Asian/PI (median household income tertile 1) are based on small sample sizes (<100 cases and/or ≤20 deaths). Due to small sample size across multiple categories, NH-NA/AN estimates are not shown in the figure.

Approximately one-third (35%) of NH-Black women had the non-endometrioid subtype, compared to less than a fifth in other racial/ethnic groups (17% are non-endometrioid among NH-White, NH-Asian/PI, and Hispanic, and 15% in NH-NA/AN women) (Figure S4). Regardless, NH-Black women with both endometrioid and non-endometrioid subtypes exhibited the poorest survival rates across all socioeconomic characteristics (Figure 3 and Figure 4). Detailed estimates are provided in the Supplementary Materials (Tables S3 and S4).

The following results present age-standardized five-year survival estimates, comparing the highest and lowest socioeconomic levels within each racial or ethnic group, rather than across different racial or ethnic groups.

Among NH-White women with the endometrioid subtype, there were small but significant survival differences between the highest (most advantaged) and lowest (least advantaged) socioeconomic levels for all four measures: %<HS education (89.3% vs. 87.8%, *p* < 0.001), %<150 percent poverty (89.4% vs. 87.5%, *p* < 0.001), %unemployment (89.5% vs. 88.5%, *p* 0.003), and median household income (89.2% vs. 87.7%, *p* = 0.018). For NH-White women with the non-endometrioid subtype, significant differences were observed for %<HS education (60.3% vs. 58.0%, *p* = 0.001) and %<150 percent poverty (60.7% vs. 57.4%, *p* = 0.002).

NH-Black women with the endometrioid subtype showed relatively larger differences between the highest and the lowest socioeconomic levels compared with other racial/ethnic groups included in this study: %<HS education (76.3% vs. 72.8%, *p* < 0.001), %<150 percent poverty (75.2% vs. 69.2%, *p* < 0.001), %unemployment (74.9% vs. 71.2%, *p* = 0.005), and median household income (74.2% vs. 68.6%, *p* < 0.001). For the non-endometrioid subtype, significant differences were observed in %<HS education (43.5% vs. 40.7%, *p* = 0.020), %<150 percent poverty (44.8% vs. 39.0%, *p* < 0.001), and median household income (42.7% vs. 38.4%, *p* = 0.003). 

Hispanic women with the endometrioid subtype also showed statistically significant differences between the highest and lowest socioeconomic levels of %<HS education (83.8% vs. 82.7%, *p* = 0.017), %<150 percent poverty (84.7% vs. 80.7%, *p* < 0.001), and median household income characteristics (83.8% vs.80.7%, <0.001), but the magnitude of these differences was small. Similarly, there were small, but significant differences among Hispanic women with non-endometrioid cancer across the highest and lowest socioeconomic levels of %<150 percent poverty (56.0% vs. 53.3%, *p* = 0.005) and %unemployment (57.7% vs. 52.9%, *p* = 0.021). 

Survival among NH-Asian/PI women in counties with second tertile median household income remained significantly lower compared to the first tertile (counties with median household income < USD 47,060) for both endometrioid and non-endometrioid subtypes, likely due to the population distribution (Figure S1). 

Among NH-NA/AN women, certain socioeconomic characteristics seem to contribute to large absolute survival differences across socioeconomic levels (Table S3). However, due to the small sample size, these findings should be interpreted with caution (Table S4).

### 3.6. Findings from Post Hoc Analyses

Findings from our first post hoc analysis show that, given the rarity of the potentially misclassified and heterogeneous ”other uterine cancers,“ the exclusion of these histologic subtypes did not impact our findings.

In our second post hoc analysis, we found significant survival differences across the highest and the lowest socioeconomic levels of %<HS education, <%150 percent poverty, %unemployment, and median household income in Northeast, South, and West/Midwest, with the largest differences between the socioeconomic levels observed in the Northeast region, followed by South (Table S5).

## 4. Discussion

### 4.1. Principal Findings 

Our findings suggest that lower county-level socioeconomic status correlates with poorer five-year relative survival rates of uterine cancer across all racial/ethnic groups and histologic subtypes. In line with our findings, other studies have shown that lower area-based socioeconomic status, serving as a proxy for individual-level socioeconomic status, is associated with poorer survival outcomes in women with uterine cancer [8,9,10,12,32,33,34]. These associations persist even when considering stage, histology, and treatment factors, with the largest disparity observed among NH-Black women [4,5,12]. 

Within strata of the various county-level socioeconomic characteristic, wide gaps in survival persisted among NH-Black compared with all other racial and ethnic groups, particularly NH-White women. More importantly, worse survival rates were observed among NH-Black women even in the most advantaged counties with the highest education levels, lowest poverty and unemployment rates, and highest incomes. Collectively, these findings suggest that county-level socioeconomic factors including level of education, poverty, unemployment, and income play a minimal role in explaining uterine cancer survival disparities among NH-Black women. 

While the proportion of women living in disadvantaged communities (communities with concentrated disadvantages according to socioeconomic determinants) tended to be highest among NH-Black, NH-NA/AN, and Hispanic women, the impact of county-level socioeconomic factors on survival rates among Hispanic women showed minimal absolute differences. Some studies show that living within ethnic enclaves may benefit the survival outcomes of people living with (any cancer, uterine and breast cancers) or without cancer of certain ethnic groups, but not all [35,36,37,38]. Data are limited and not consistent regarding the impact of ethnic enclaves on the survival outcomes of Hispanic/Latina women by socioeconomic status [35,37]. Further investigations are needed at the individual level, particularly among Hispanic women with uterine cancer. 

Our post hoc analysis by geographic regions shows that the correlation between lower county-level socioeconomic status and poorer five-year relative survival rates of uterine cancer persists across different geographic regions. Additionally, our analysis revealed greater survival disparities across socioeconomic characteristics in the Northeast region, followed by the South region. 

### 4.2. Socioeconomic Status and Survival Differences by Histology 

While we did not observe strong differences in the influence of county-level socioeconomic characteristics on survival rates of women with uterine cancer by histology, it is worth noting that the impact of these measures appeared to be slightly greater for women with endometrioid histology. Because endometrioid cancers are treated surgically and highly curable, it is plausible that socioeconomic barriers related to treatment and healthcare access may have a greater influence on survival rates, whereas non-endometrioid cancers are less amenable to curative treatment and have worse outcomes in general.

NH-Black women consistently exhibit a survival gap compared to NH-White women, across both endometrioid and non-endometrioid subtypes. Despite a higher proportion being diagnosed with the aggressive non-endometrioid subtype, this disparity persists even after adjusting for clinical, socioeconomic, and treatment-related factors [5,39]. A mediation analysis found that clinicopathologic factors contributed most significantly to survival differences in NH-Black women with non-endometrioid uterine cancer compared to NH-White women (about 40%), followed by sociodemographic (about 9%) and treatment factors (about 7%) [39]. Despite adjustments, a persistent survival gap in NH-Black women remained, indicating unknown and unaccounted factors contribute to survival disparities in NH-Black women [39]. Histology differences in Black women with uterine cancer may reflect a complex interplay between multiple factors, possibly impacted by chronic lifestyle and socioeconomic stressors [40]. However, these complex associations, particularly by histology, remain poorly understood and are beyond the scope of this study. 

### 4.3. Complex Interplay between Socioeconomic Characteristics, Race/Ethnicity, and Survival

We found that NH-Black women had worse survival rates compared to other racial/ethnic groups across all socioeconomic levels, regardless of histology and stage. The complex interplay between social determinants of health, systemic racism and discrimination, and race/ethnicity in regard to uterine cancer goes beyond our selected socioeconomic characteristics [20]. Instead, it encompasses the confluence of all these factors, particularly for Black women [15,33,40], which may pose challenges in interpreting county-level analyses but are crucial for comprehensive understanding and addressing disparities. For example, residential segregation [20,41] and the systematic distribution of risk factors [13,14] and healthcare resources [11] intentionally varies across race/ethnicity and socioeconomic levels, collectively influencing chronic comorbidities [15], and ultimately contributing to survival outcomes [11,13,14]. One study linked marginalization, including material deprivation factors like education, income, and unemployment, to advanced-stage diagnosis in women with uterine cancer, even after adjusting for age, obesity, comorbidities, and histology [19]. Another found either race/ethnicity or income, but not both, was associated with advanced-stage disease [33]. Both studies show a tight link (collinearity) between race and socioeconomic factors, highlighting the challenge in determining their independent impact [19,33]. 

With no standard screening protocols, uterine cancer diagnosis relies on symptomatic presentation (e.g., abnormal bleeding, abnormal imaging), individual health-seeking behaviors, and accurate, timely, and unbiased diagnostic evaluation by healthcare providers. Delays in any one of these steps may be associated with more advanced disease and worse outcomes [42]. NH-Black women in particular report delayed symptom appraisal, health-seeking behaviors, and encounter poor-quality healthcare such as less likelihood of receiving of guideline concordant diagnostic work-up and treatment, symptom dismissals, and delays in diagnosis and treatment [11,42,43,44]. Health literacy, often tied to education level, likely influences symptom recognition and, when combined with poverty and unemployment, may exacerbate barriers to accessing care. Racism, experienced through discrimination and residential segregation, often lead to socioeconomic disparities, limiting access to education and job opportunities [20]. These disparities can result in variations in neighborhood environments, such as access to healthy food, quality healthcare, and exposure to lifetime stressors [20,45,46], ultimately increasing the risk of morbidity and mortality [20,47]. These health inequalities are likely exacerbated by a lack of understanding of risk factors, early diagnostic factors, and prognostic factors associated with aggressive non-endometrioid subtypes, which are more common among NH-Black women [11,47,48]. 

### 4.4. Strengths, Limitations, and Future Directions

In our study, we offer a unique comparison of age-standardized five-year relative survival estimates of uterine cancer across various socioeconomic categories by race/ethnicity, encompassing major histologic subtypes. Such comprehensive analyses are scarce in the existing literature, underscoring the novelty and importance of our research. We calculated age-standardized survival estimates that facilitate comparisons across socioeconomic and racial/ethnic groups by mitigating differences in age distribution across groups [49], as well as the independent effects of age on outcomes [50]. We utilized data from 20 population-based cancer registries collectively covering 42% of the U.S. population based on the 2020 U.S. Census [21]. SEER provides a large and diverse sample of women diagnosed with uterine cancer with comprehensive histology, stage at diagnosis, and survival data. Moreover, through linkage with the ACS database and the U.S. Census, it offers information on county-level socioeconomic estimates including education, poverty, unemployment, income, and information on county-level urban population density. 

While U.S.-level statistics generated using SEER data and the overall quality of the SEER database have been well established [1], we acknowledge certain limitations pertaining to its use in our current analysis [51]. Limitations of our study include the absence of key information on potential confounders (e.g., underreported/incomplete adjuvant therapy data, missing stage at diagnosis prior to 2004, and missing/incomplete data on tumor grade, comorbidities, and other risk factors) and possible misclassification of rare histologic subtypes of uterine cancer which would not have impacted our main findings. Moreover, individual migrations from registry catchment area to an outside area can result in loss to follow-up and misclassification of the county-level socioeconomic status, because socioeconomic characteristics were determined based on the lived-in area at the time of diagnosis [51]. Because each county-level socioeconomic characteristic is based on different combinations of counties that fall within a certain tertile level, we cannot rule out the influence of other geographic factors that may contribute to survival. 

Our findings suggest a link between county-level socioeconomic factors and uterine cancer survival across racial/ethnic groups and minimal impact of socioeconomic factors on survival across histology; future investigations are warranted to include factors beyond traditionally considered socioeconomic indicators such as education, poverty, unemployment, and income, and inclusion of different research methods to identify root causes. It is important to note that our conclusions are limited to elucidating the link between county-level socioeconomic factors and overall survival rates in an ecological analysis. Racial/ethnic groups within the same geographic area may experience different socioeconomic conditions and individual-level challenges, which may have a larger impact on survival across racial/ethnic groups than county-level socioeconomic status alone. Moreover, a multivariable analysis that accounts for major confounders at individual level (e.g., treatment type and receipt of guideline concordant therapy, comorbidities, experiences of discrimination and other chronic lifetime stressors), in conjunction with a more refined geographical area-based socioeconomic status measure (defined at the zip code level) of, may offer a more precise understanding of the relationship between socioeconomic status and survival. 

Considering the persistent survival disparities among underrepresented racial/ethnic groups, especially NH-Black women, which may worsen due to increasing incidence rates of aggressive histologic subtypes, it is crucial to initially identify and better understand modifiable socioeconomic determinants influencing these survival disparities. 

## 5. Conclusions

We observed significant associations of lower county-level socioeconomic characteristics with reduced age-adjusted five-year relative survival rates, both overall and among different racial/ethnic groups. County-level socioeconomic characteristics had a minimal impact on survival rates across histologic subtypes. Regardless of the county-level socioeconomic level and tumor characteristics, NH-Black women consistently show worse survival outcomes compared to other racial/ ethnic groups studied in our analysis. 

Our findings further our understanding of the relationship between selected county-level socioeconomic factors and survival inequalities among different racial/ethnic groups. Although reducing socioeconomic inequities could potentially have an impact on survival outcomes across racial and ethnic groups, it is clear from our findings and from other individual-level studies [8,9,10] that more research is needed to better understand reasons for persistent uterine cancer survival disparities among NH-Black women beyond these county-level socioeconomic characteristics. Future studies are warranted to investigate the relationship between social determinants of health that go beyond the traditional socioeconomic characteristics (county or individual level), including systemic racism, discrimination, exposure to chronic lifetime stressors and resilience, and survival outcomes, especially among NH-Black women [6,7,8].

## Figures and Tables

**Table 1:** Age standardized five-year relative survival rates (2000–2019) of women diagnosed with microscopically confirmed uterine cancer overall and across demographic, clinical, and county-level socioeconomic characteristics, among U.S. women aged ≥ 30 Years, diagnosed between the years 2000 and 2018, in SEER 22.

Population Characteristics	Cases, N (%)	Deaths, N	Survival Rate (95% CI) ^a^	SRR	*p*-Value ^b^
Overall, All Women	333,013	79,782	77.7 (77.5, 77.9)	-	-
Race/Ethnicity					
NH-White	229,273 (68.85)	51,882	81.2 (80.9, 81.4)	1.00	Reference
NH-Black	34,200 (10.27)	14,096	57.7 (57.0, 58.4)	0.71	<0.001
NH-Asian/PI	21,209 (6.37)	3890	75.8 (74.8, 76.8)	0.93	0.0908
NH-NA/AN	1615 (0.48)	338	77.1 (72.6, 81.0)	0.95	0.0673
Hispanic	44,716 (13.43)	9576	73.1 (72.4, 73.8)	0.90	<0.001
Histology					
Endometrioid	247,240 (74.69)	39,919	87.1 (86.8, 87.3)	1.00	Reference
Non-Endometrioid	61,620 (18.62)	28,525	55.7 (55.3, 56.2)	0.63	<0.001
Sarcoma	14,610 (4.41)	7060	44.4 (43.2, 45.7)	0.51	<0.001
Other	7543 (2.28)	4278	42.2 (41.0, 43.5)	0.48	<0.001
Stage at Diagnosis ^c^					
Localized	184,719 (69.68)	20,751	93.0 (92.7, 93.2)	1.00	Reference
Regional	55,805 (21.05)	19,463	64.6 (64.1, 65.1)	0.69	<0.001
Distant	24,583 (9.27)	19,475	16.6 (16.1, 17.2)	0.18	<0.001
%<HS Education ^d^					
Tertile 1	96,211 (29.07)	21,193	80.2 (79.8, 80.6)	1.00	Reference
Tertile 2	119,901 (36.23)	28,580	78.3 (78.0, 78.7)	0.98	<0.001
Tertile 3	114,873 (34.70)	30,000	74.7 (74.3, 75.1)	0.93	<0.001
%<150 Percent Poverty ^d^					
Tertile 1	149,375 (45.13)	33,111	79.8 (79.5, 80.2)	1.00	Reference
Tertile 2	131,906 (39.85)	32,518	77.0 (76.6, 77.3)	0.96	<0.001
Tertile 3	49,704 (15.02)	14,144	72.9 (72.3, 73.5)	0.91	<0.001
%Unemployment ^d^					
Tertile 1	45,981 (13.89)	10,014	80.5 (79.9, 81.1)	1.00	Reference
Tertile 2	166,282 (50.24)	39,305	78.3 (78.0, 78.6)	0.97	<0.001
Tertile 3	118,722 (35.87)	30,454	75.7 (75.3, 76.0)	0.94	<0.001
Median Household Income (USD) ^d^					
Tertile 1	28,623 (8.65)	8103	73.3 (72.5, 74.1)	1.00	Reference
Tertile 2	43,064 (13.01)	10,620	78.3 (77.7, 78.9)	1.07	<0.001
Tertile 3	259,298 (78.34)	61,050	78.1 (77.8, 78.3)	1.07	<0.001
% Urban ^d^					
Tertile 1	9019 (2.72)	2237	77.8 (76.4, 79.1)	1.00	Reference
Tertile 2	30,149 (9.11)	7302	79.0 (78.3, 79.7)	1.02	0.93385
Tertile 3	291,819 (88.17)	70,234	77.5 (77.3, 77.8)	1.00	0.11071

Notes. Percentages were calculated using non-missing data. Abbreviations: CI, confidence interval; NA/AN, Native American/ Alaska Native; NH, non-Hispanic; PI, Pacific Islander; SES, socioeconomic status; SRR, survival rate ratio. ^a^ First matching primary uterine cancer cases (diagnosed 2000–2018) were included, and survival assessed based on all-cause mortality estimated through 2019. ^b^ *p*-values (two tailed) indicates the statistical difference between two 5-year relative survival estimates computed from the z-statistic with the formula *p* = 2 × (1 − Φ(∣z∣)), where Φ(x) = the standard normal distribution’s cumulative distribution function, and ∣z∣ = absolute z-score. ^c^ Stage at diagnosis was not available prior to 2004, resulting in a total of n = 54,918 missing data points. Additionally, we set n = 10,978 (unknown/not-staged), and n = 10 (in situ) tumors as missing. ^d^ All county-level SES characteristics, except %urban (2010 U.S. Census County Attributes), were sourced from the ACS database (2015–2019) linked to SEER 22. The following ranges were used for SES tertile cutoffs: %<HS Education (1.12% to 73.56%; T1 < 9.48%, T2 9.48–14.74%, and T3 > 14.74%), %<150 Poverty (4.84% to 68.30%; T1 < 20.92%, T2 20.92–28.12%, and T3 > 28.12%), %Unemployed (0% to 27.15%; T1 < 4.07%, T2 4.07–5.86%, T3 > 5.86%), Median Household Income (USD 21,500 to USD 142,300; T1 < USD 47,060, T2 USD 47,060 to USD 56,590, T3 > USD 56,590), and %urban (0 to 100% urban; T1 < 24.53%, T2 24.53–57.02%, T3 > 57.02%).

## Data Availability

All data utilized in this study are de-identified and are publicly available. Data can be accessed at https://seer.cancer.gov/ (accessed on 8 April 2024).

## Supplementary Materials

The following supporting information can be downloaded at: https://www.mdpi.com/article/10.3390/cancers16152747/s1, Figure S1. Proportion of eligible women diagnosed with uterine cancer by socioeconomic characteristics across race/ethnicity. Figure S2. Race/ethnicity specific age-standardized 5-year survival rates by histologic subtype and stage among women diagnosed with uterine cancer between 2000 and 2018, with survival assessed through 2019. Figure S3. Race/ethnicity specific age-standardized 5-year survival rates by socioeconomic characteristics and stage at diagnosis among women diagnosed with uterine cancer between 2000 and 2018, with survival assessed through 2019. Figure S4. Proportion of women diagnosed with uterine cancer by histologic subtypes and stage at diagnosis, across race/ethnicity. Table S1. Number of women diagnosed with uterine cancer cases by histologic subtype among U.S. women aged ≥ 30 years, SEER 22 (2000–2018). Table S2. Overall five-year relative survival rates (not standardized for age, 2000–2019) of women diagnosed with uterine cancer across demographic, clinical, and socioeconomic characteristics, among U.S. women aged ≥ 30 years, diagnosed between the years 2000 and 2018, SEER 22. Table S3. Race/ethnicity specific age-standardized five-year relative survival rates (2000–2019) of women diagnosed with uterine cancer across socioeconomic characteristics, overall and by major histologic subtypes (endometrioid and non-endometrioid cancer) among U.S. women aged ≥ 30 years, diagnosed between the years 2000 and 2018, SEER 22. Table S4. Number of women diagnosed with uterine cancer and deaths across race/ethnicity, socioeconomic characteristics, and histology, among U.S. women aged ≥ 30 years, diagnosed between the years 2000 and 2018, SEER 22. Table S5. Age-standardized five-year relative survival rates (2000–2019) of women diagnosed with uterine cancer by U.S. geographic region and socioeconomic characteristics among U.S. women aged ≥ 30 years, diagnosed between the years 2000 and 2018, SEER 22.

## References

[1] Siegel R.L., Giaquinto A.N., Jemal A. (2024). Cancer statistics 2024. CA Cancer J. Clin..

[2] Clarke M.A., Devesa S.S., Harvey S.V., Wentzensen N. (2019). Hysterectomy-Corrected Uterine Corpus Cancer Incidence Trends and Differences in Relative Survival Reveal Racial Disparities and Rising Rates of Nonendometrioid Cancers. J. Clin. Oncol..

[3] Bain R.P., Greenberg R.S., Chung K.C. (1987). Racial differences in survival of women with endometrial cancer. Am. J. Obstet. Gynecol..

[4] Baskovic M., Lichtensztajn D.Y., Nguyen T., Karam A., English D.P. (2018). Racial disparities in outcomes for high-grade uterine cancer: A California cancer registry study. Cancer Med..

[5] Bregar A.J., Rauh-Hain J.A., Spencer R., Clemmer J.T., Schorge J.O., Rice L.W., Del Carmen M.G. (2017). Disparities in receipt of care for high-grade endometrial cancer: A National Cancer Data Base analysis. Gynecol. Oncol..

[6] Felix A.S., Cohn D.E., Brasky T.M., Zaino R., Park K., Mutch D.G., Creasman W.T., Thaker P.H., Walker J.L., Moore R.G. (2018). Receipt of adjuvant endometrial cancer treatment according to race: An NRG Oncology/Gynecologic Oncology Group 210 Study. Am. J. Obstet. Gynecol..

[7] Giaquinto A.N., Miller K.D., Tossas K.Y., Winn R.A., Jemal A., Siegel R.L. (2022). Cancer statistics for African American/Black People 2022. CA Cancer J. Clin..

[8] Donkers H., Bekkers R., Massuger L., Galaal K. (2019). Systematic review on socioeconomic deprivation and survival in endometrial cancer. Cancer Causes Control.

[9] Njoku K., Barr C.E., Hotchkies L., Quille N., Wan Y.L., Crosbie E.J. (2021). Impact of socio-economic deprivation on endometrial cancer survival in the North West of England: A prospective database analysis. Br. J. Obstet. Gynaecol..

[10] Zadnik V., Žagar T., Tomšič S., Mihor A., Lokar K. (2022). Cancer Patients’ Survival According to Socioeconomic Environment in a High-Income Country with Universal Health Coverage. Cancers.

[11] Schlumbrecht M., Wright K., George S. (2023). Unique Considerations in Early Detection, Risk, and Awareness of Endometrial Cancer in Black Women. Cancer Control.

[12] Whetstone S., Burke W., Sheth S.S., Brooks R., Cavens A., Huber-Keener K., Scott D.M., Worly B., Chelmow D. (2022). Health Disparities in Uterine Cancer: Report from the Uterine Cancer Evidence Review Conference, USA. Obstet. Gynecol..

[13] Sarink D., Wilkens L.R., White K.K., Le Marchand L., Wu A.H., Setiawan V.W., Park S.L., Park S.Y., Killeen J.L., Merritt M.A. (2021). Racial/ethnic differences in anthropometric and hormone-related factors and endometrial cancer risk: The Multiethnic Cohort Study. Br. J. Cancer.

[14] Donkers H., Bekkers R., Massuger L.F., Galaal K. (2020). Socioeconomic deprivation and survival in endometrial cancer: The effect of BMI. Gynecol. Oncol..

[15] Sonderlund L.A., Charifson M., Schoenthaler A., Carson T., Williams N.J. (2022). Racialized economic segregation and health outcomes: A systematic review of studies that use the Index of Concentration at the Extremes for race, income, and their interaction. PLoS ONE.

[16] Adamkiewicz G., Zota A.R., Fabian M.P., Chahine T., Julien R., Spengler J.D., Levy J.I. (2011). Moving environmental justice indoors: Understanding structural influences on residential exposure patterns in low-income communities. Am. J. Public Health.

[17] Collins T.W., Grineski S.E., Shaker Y., Mullen C.J. (2022). Communities of color are disproportionately exposed to long-term and short-term PM. Environ. Res..

[18] Josey K.P., Delaney S.W., Wu X., Nethery R.C., DeSouza P., Braun D., Dominici F. (2023). Air Pollution and Mortality at the Intersection of Race and Social Class. N. Engl. J. Med..

[19] Helpman L., Pond G.R., Elit L., Anderson L.N., Seow H. (2020). Endometrial cancer presentation is associated with social determinants of health in a public healthcare system: A population-based cohort study. Gynecol. Oncol..

[20] Popescu I., Duffy E., Mendelsohn J., Escarce J.J. (2018). Racial residential segregation, socioeconomic disparities, and the White-Black survival gap. PLoS ONE.

[21] Surveillance, Epidemiology and End Results Program (SEER) SEER*Stat Database: Incidence—SEER Research Plus Data, 18 Registries, Nov 2020 Sub (2000–2018)—Linked To County Attributes—Total U.S., 1969–2019 Counties, National Cancer Institute, DCCPS, Surveillance Research Program, Released April 2021 (November 2020 Submission). www.seer.cancer.gov.

[22] Clarke M.A., Devesa S.S., Hammer A., Wentzensen N. (2022). Racial and ethnic differences in hysterectomy-corrected uterine corpus cancer mortality by stage and histologic subtype. JAMA Oncol..

[23] Surveillance, Epidemiology and End Results Program (SEER) Race and Hispanic Ethnicity Changes (November 2021 Submission). https://seer.cancer.gov/seerstat/variables/seer/race_ethnicity/.

[24] Gomez S.L., Le G.M., West D.W., Satariano W.A., O’Connor L. (2003). Hospital policy and practice regarding the collection of data on race, ethnicity, and birthplace. Am. J. Public Health.

[25] Surveillance, Epidemiology and End Results Program (SEER) Localized/Regional/Distant Stage Adjustments 2023. https://seer.cancer.gov/seerstat/variables/seer/yr1975_2020/lrd_stage/index.html#footnotea.

[26] Surveillance, Epidemiology and End Results Program (SEER) Summary Stage Manual—Female Genital System 2000. https://seer.cancer.gov/tools/ssm/ssm2000/breast_femgen.pdf.

[27] Berkowitz S.A., Traore C.Y., Singer D.E., Atlas S.J. (2015). Evaluating area-based socioeconomic status indicators for monitoring disparities within health care systems: Results from a primary care network. Health Serv. Res..

[28] Surveillance, Epidemiology and End Results Program (SEER). SEER*Stat Rate Exercise 5: Incidence Rates by County Attributes. https://seer.cancer.gov/seerstat/tutorials/rate5/webprint/.

[29] Mariotto A.B., Zou Z., Johnson C.J., Scoppa S., Weir H.K., Huang B. (2018). Geographical, racial and socio-economic variation in life expectancy in the US and their impact on cancer relative survival. PLoS ONE.

[30] Centers for Disease Control and Prevention (CDC), Division of Cancer Prevention and Control Suppression of Rates and Counts (Published 2023). https://www.cdc.gov/cancer/uscs/technical_notes/stat_methods/suppression.htm.

[31] U.S. Department of Commerce Economics and Statistics Administration U.S. Census Bureau Census Regions and Divisions of the United States 2013. https://www2.census.gov/geo/pdfs/maps-data/maps/reference/us_regdiv.pdf.

[32] Snider N.G., Hastert T.A., Nair M., Kc M., Ruterbusch J.J., Schwartz A.G., Peters E.S., Stoffel E.M., Rozek L.S., Purrington K.S. (2023). Area-level Socioeconomic Disadvantage and Cancer Survival in Metropolitan Detroit. Cancer Epidemiol. Biomark. Prev..

[33] Madison T., Schottenfeld D., James S.A., Schwartz A.G., Gruber S.B. (2004). Endometrial cancer: Socioeconomic status and racial/ethnic differences in stage at diagnosis, treatment, and survival. Am. J. Public Health.

[34] Cheung M.R. (2013). African American race and low income neighborhoods decrease cause specific survival of endometrial cancer: A SEER analysis. Asian Pac. J. Cancer Prev..

[35] Von Behren J., Abrahão R., Goldberg D., Gomez S.L., Setiawan V.W., Cheng I. (2018). The influence of neighborhood socioeconomic status and ethnic enclave on endometrial cancer mortality among Hispanics and Asian Americans/Pacific Islanders in California. Cancer Causes Control.

[36] Yang T.C., Zhao Y., Song Q. (2017). Residential segregation and racial disparities in self-rated health: How do dimensions of residential segregation matter?. Soc. Sci. Res..

[37] Shariff-Marco S., Gomez S.L., Canchola A.J., Fullington H., Hughes A.E., Zhu H., Pruitt S.L. (2020). Nativity, ethnic enclave residence, and breast cancer survival among Latinas: Variations between California and Texas. Cancer.

[38] Cha J., Bustamante G., Lê-Scherban F., Duprez D., Pankow J.S., Osypuk T.L. (2023). Ethnic enclaves and incidence of cancer among US ethnic minorities in the multi-ethnic study of atherosclerosis. J. Racial Ethn. Health Disparities.

[39] Karia P.S., Huang Y., Tehranifar P., Wright J.D., Genkinger J.M. (2023). Racial and ethnic differences in type II endometrial cancer mortality outcomes: The contribution of sociodemographic, clinicopathologic, and treatment factors. Gynecol. Oncol..

[40] Doll K.M. (2018). Investigating Black-White disparities in gynecologic oncology: Theories, conceptual models, and applications. Gynecol. Oncol..

[41] Williams D.R., Collins C. (2001). Racial residential segregation: A fundamental cause of racial disparities in health. Public Health Rep..

[42] Doll K.M., Khor S., Odem-Davis K., He H., Wolff E.M., Flum D.R., Ramsey S.D., Goff B.A. (2018). Role of bleeding recognition and evaluation in Black-White disparities in endometrial cancer. Am. J. Obstet. Gynecol..

[43] Doll K.M., Khor S., Odem-Davis K., He H., Wolff E.M., Flum D.R., Ramsey S.D., Goff B.A. (2020). Assessment of Prediagnostic Experiences of Black Women with Endometrial Cancer in the United States. JAMA Netw. Open.

[44] Barrington D.A., Sinnott J.A., Nixon D., Padamsee T.J., Cohn D.E., Doll K.M., Donneyong M.M., Felix A.S. (2022). More than treatment refusal: A National Cancer Database analysis of adjuvant treatment refusal and racial survival disparities among women with endometrial cancer. Am. J. Obstet. Gynecol..

[45] Moore J.X., Andrzejak S.E., Bevel M.S., Jones S.R., Tingen M.S. (2022). Exploring racial disparities on the association between allostatic load and cancer mortality: A retrospective cohort analysis of NHANES, 1988 through 2019. SSM Popul. Health.

[46] Moore J.X., Bevel M.S., Aslibekyan S., Akinyemiju T. (2021). Temporal changes in allostatic load patterns by age, race/ethnicity, and gender among the US adult population; 1988–2018. Prev. Med..

[47] Hicks M.L., Hicks M.M., Mathews R.P., Khabele D., Clare C.A., Balogun O., Lawson Y.R., Tillman R.H., Butler R., Spann C.O. (2024). Racial disparities in endometrial cancer: Where are we after 26 years?. Gynecol. Oncol..

[48] Pinheiro P.S., Medina H., Callahan K.E., Kwon D., Ragin C., Sherman R., Kobetz E.N., Jemal A. (2020). Cancer mortality among US blacks: Variability between African Americans, Afro-Caribbeans, and Africans. Cancer Epidemiol..

[49] Rabe M., Exploring the Racial and Ethnic Diversity of Various Age Groups 2023: United States Census Bureau. https://www.census.gov/newsroom/blogs/random-samplings/2023/09/exploring-diversity.html.

[50] Wakkerman F.C., Wu J., Putter H., Jurgenliemk-Schulz I.M., Jobsen J.J., Lutgens L.C., Haverkort M.A., de Jong M., Mens J.W.M., Wortman B.G. (2024). Prognostic impact and causality of age on oncological outcomes in women with endometrial cancer: A multimethod analysis of the randomised PORTEC-1, PORTEC-2, and PORTEC-3 trials. Lancet Oncol..

[51] Yu B.J., Gross C.P., Wilson L.D., Smith B.D. (2009). NCI SEER public-use data: Applications and limitations in oncology research. Oncology.

