# Supplementary material for: Five-Year Relative Survival Rates of Women Diagnosed with Uterine Cancer by County-Level Socioeconomic Status Overall and across Histology and Race/Ethnicity
Source: Cancers (Basel). 2024 Aug 1;16(15):2747. doi: 10.3390/cancers16152747 (PMC11311553; doi:10.3390/cancers16152747)
Supplement: Supplementary file 1 [file cancers-16-02747-s001.zip › cancers-3051665-supplementary.pdf]

Supplemental Section

Five-year Relative Survival Rates of Women Diagnosed with Uterine Cancer by County-Level Socioeconomic Status Overall and Across Histology and Race/Ethnicity

Akemi T. Wijayabahu [1,*], Jennifer K. McGee-Avila[1], Meredith S. Shiels[1], Alfonsus Adrian H. Harsono[2], Rebecca C. Arend[2] and Megan A. Clarke [1]

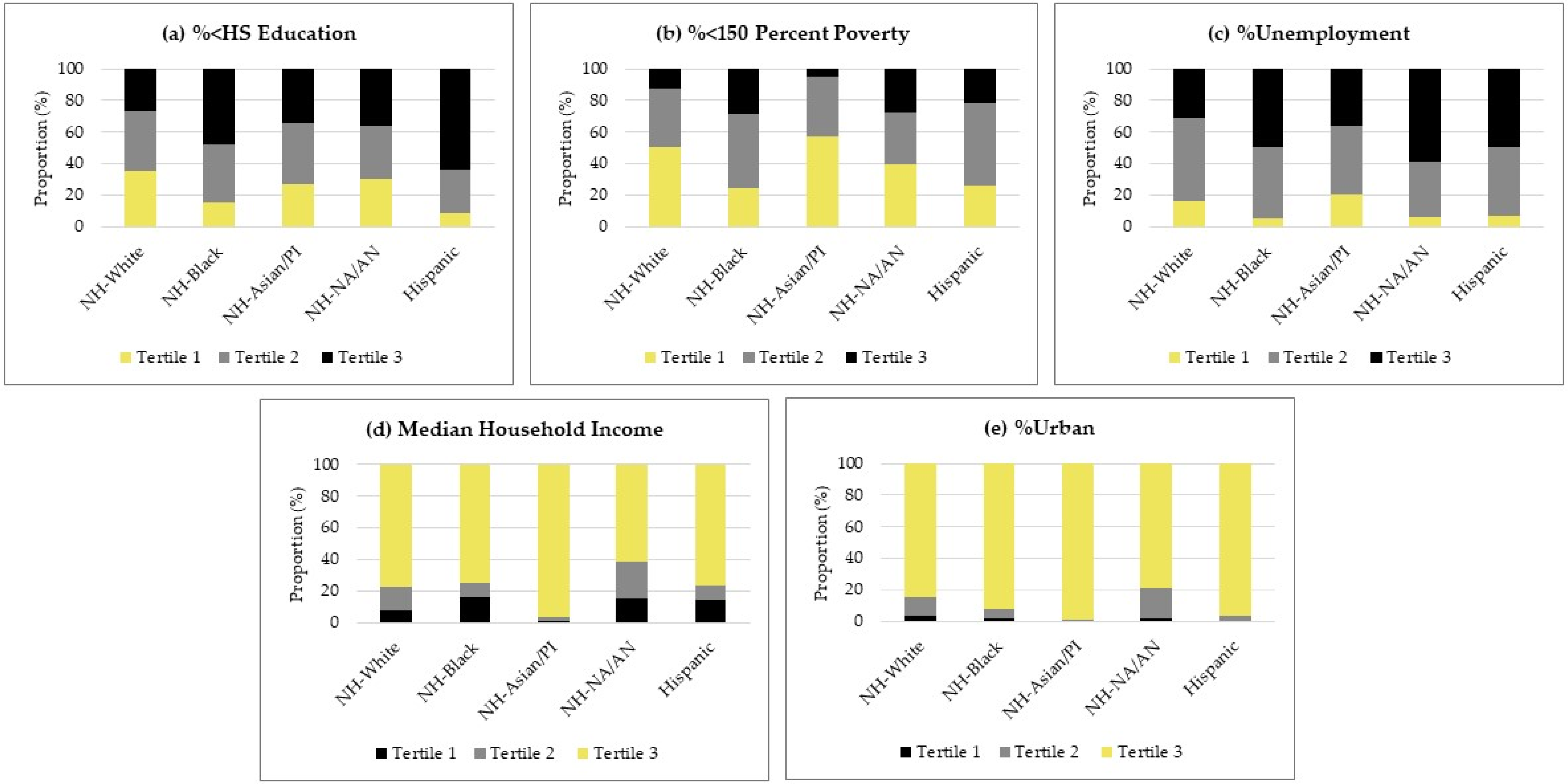

Figure S1. Proportion of Eligible Women Diagnosed with Uterine Cancer by Socioeconomic Characteristics across Race/Ethnicity.

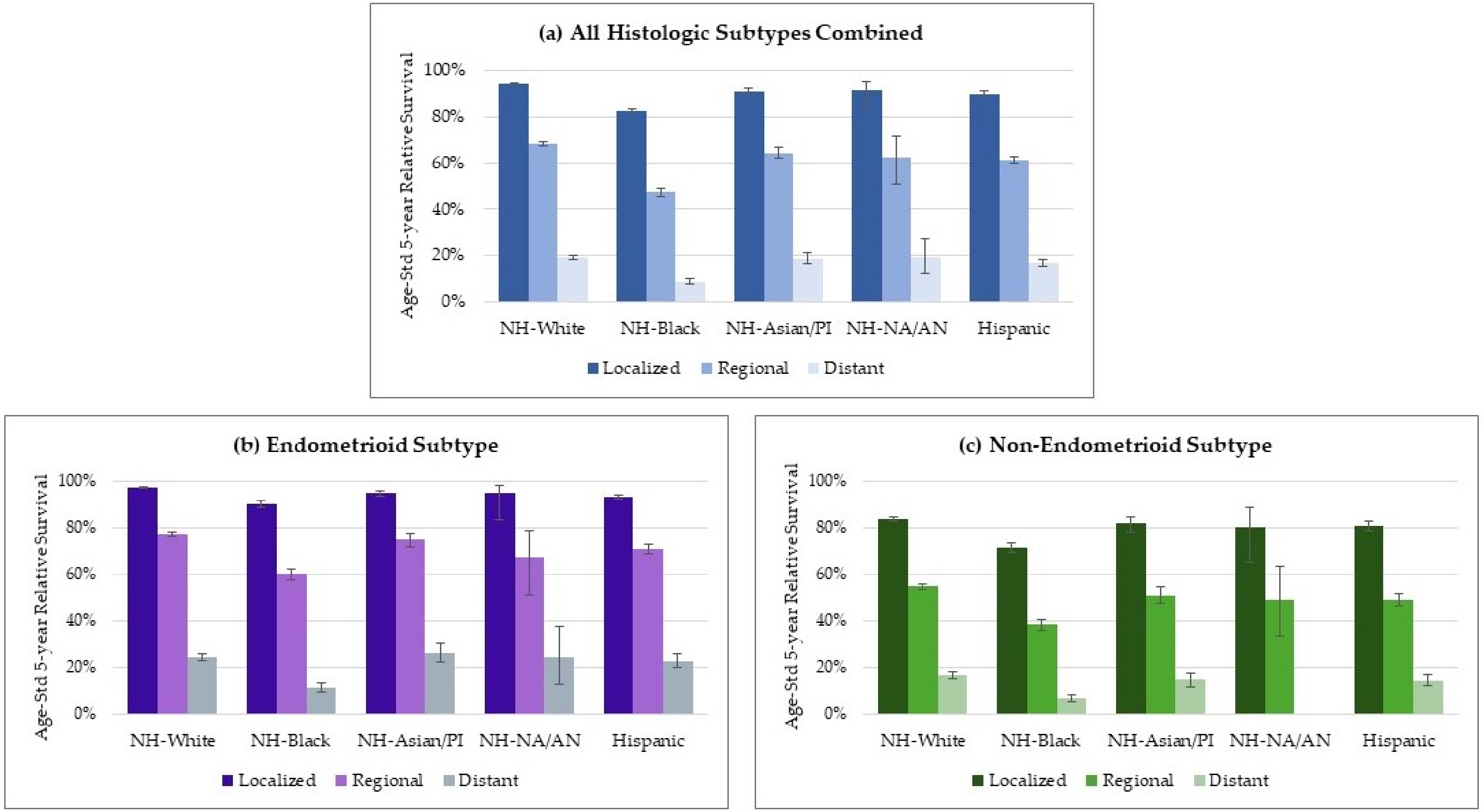

Figure S2. Race/Ethnicity Specific Age-Standardized 5-Year Survival Rates by Histologic Subtype and Stage among Women Diagnosed with Uterine Cancer between 2000 to 2018, with Survival Assessed Through 2019.

Survival estimates for NH-NA/AN distant non-endometrioid histologic subtype could not be computed.

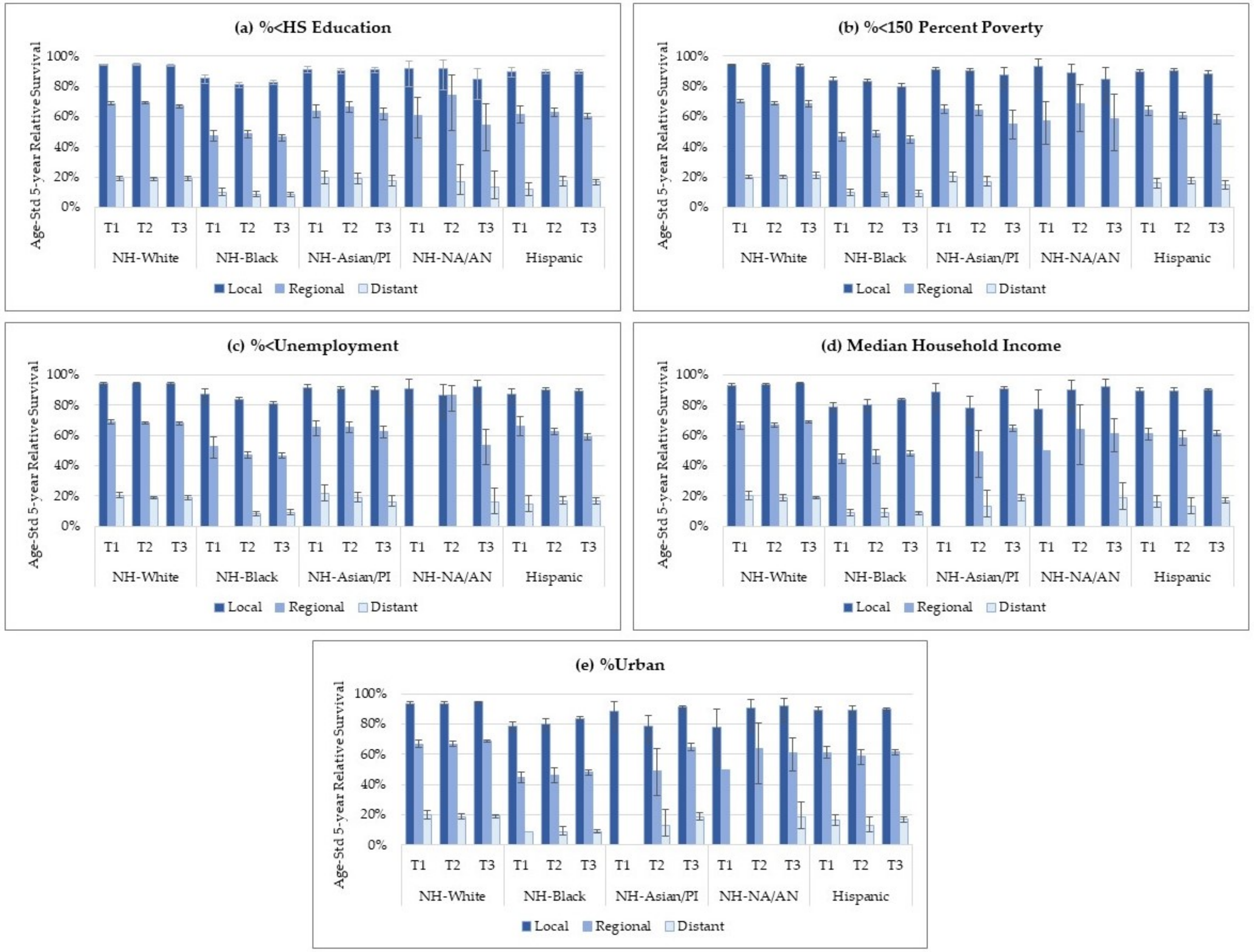

Figure S3. Race/Ethnicity Specific Age-Standardized 5-Year Survival Rates by Socioeconomic Characteristics and Stage at Diagnosis among Women Diagnosed with Uterine Cancer between 2000 to 2018, with Survival Assessed Through 2019.

Abbreviation: Tertile 1 (T1), Tertile 2 (T2) and Tertile 3 (T3). Some survival estimates for NH-Asian/PI and NH-NA/AN could not be computed.

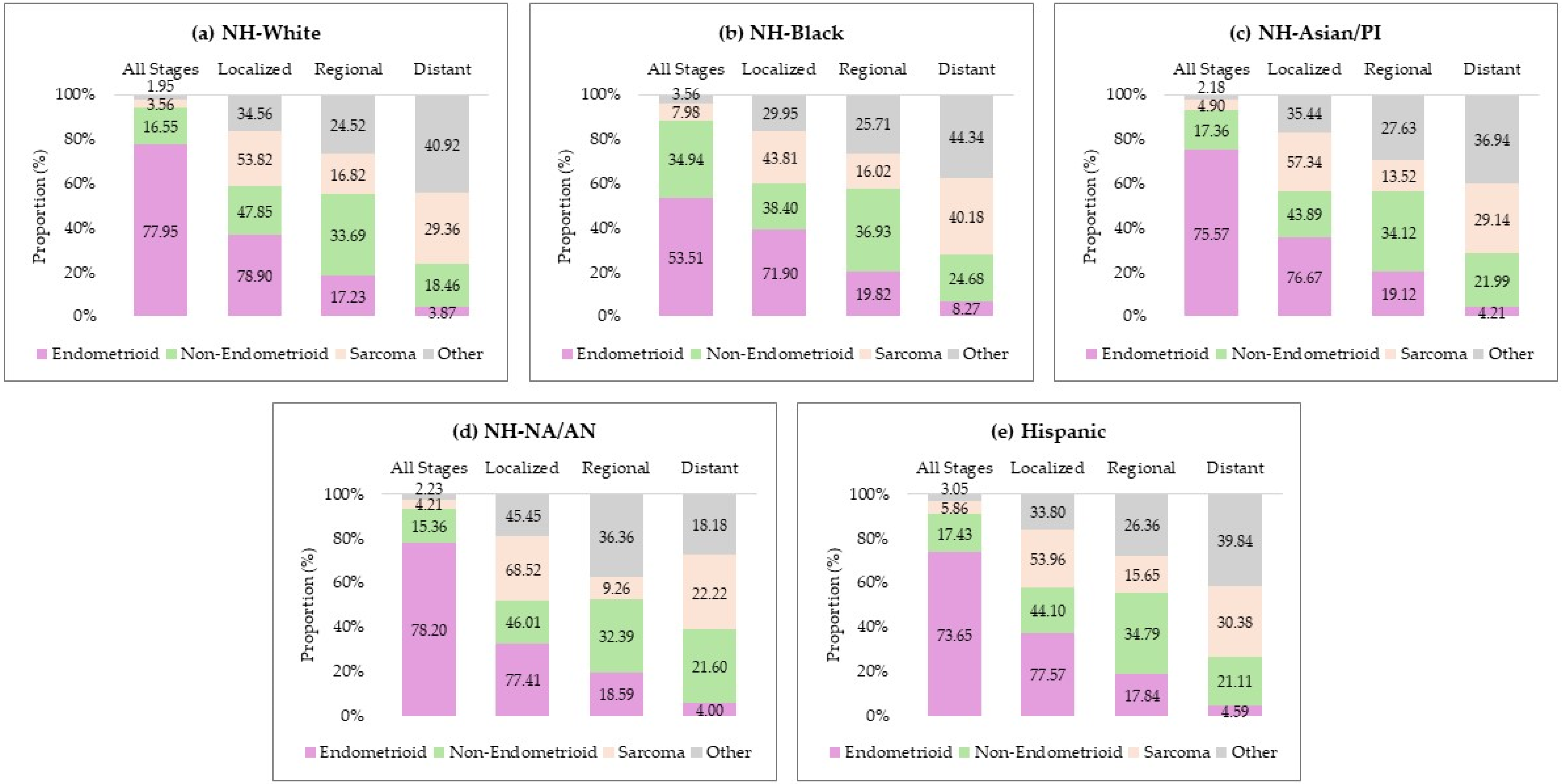

Figure S4. Proportion of Women Diagnosed with Uterine Cancer by Histologic Subtypes and Stage at Diagnosis, across Race/Ethnicity.

Table S1. Number of Women Diagnosed with Uterine Cancer Cases by Histologic Subtype Among U.S. Women Aged ≥30 Years, SEER 22 (2000-2018)

Histologic Classification [a]	ICD-O-3 Histologic Code	n	% [b]
Endometrioid Cancer (n=247,240; 74.69%)	8380/3: Endometrioid carcinoma	206864	83.67
	**8140/3: Adenocarcinoma, NOS [c]**	**29464**	**11.92**
	8570/3: Adenocarcinoma with squamous metaplasia	2852	1.15
	8480/3: Mucinous adenocarcinoma	2337	0.95
	8560/3: Adenosquamous carcinoma	2198	0.89
	8210/3: Adenocarcinoma in adenomatous polyp	1089	0.44
	8260/3: Papillary adenocarcinoma, NOS	840	0.34
	8382/3: Endometrioid adenocarcinoma, secretory variant	541	0.22
	8263/3: Adenocarcinoma in tubulovillous adenoma	284	0.11
	8050/3: Papillary carcinoma, NOS	186	0.08
	8481/3: Mucin-producing adenocarcinoma	174	0.07
	8383/3: Endometrioid adenocarcinoma, ciliated cell variant	152	0.06
	8262/3: Villous adenocarcinoma	126	0.05
	8381/3: Endometrioid adenofibroma, malignant	75	0.03
	8440/3: Cystadenocarcinoma, NOS	18	0.01
	8490/3: Signet ring cell carcinoma	17	0.01
	8141/3: Scirrhous adenocarcinoma	6	<0.01
	8261/3: Adenocarcinoma in villous adenoma	6	<0.01
	8471/3: Papillary mucinous cystadenocarcinoma	5	<0.01
	8470/3: Mucinous cystadenocarcinoma, NOS	3	<0.01
	8211/3: Tubular adenocarcinoma	2	<0.01
	8571/3: Adenocarcinoma with cartilaginous and osseous metaplasia	1	<0.01
Non-Endometrioid Cancer (n=61,620; 18.62%)	8323/3: Mixed cell adenocarcinoma	16426	26.66
	8441/3: Serous cystadenocarcinoma, NOS	12437	20.18
	8980/3: Carcinosarcoma, NOS	10270	16.67
	8460/3: Papillary serous cystadenocarcinoma	8546	13.87
	8950/3: Mullerian mixed tumor	6280	10.19
	8310/3: Clear cell adenocarcinoma, NOS	4678	7.59
	8255/3: Adenocarcinoma with mixed subtypes	1181	1.92
	8461/3: Serous surface papillary carcinoma	1066	1.73

	8951/3: Mesodermal mixed tumor	720	1.17
	8981/3: Carcinosarcoma, embryonal	16	0.03
Sarcoma (n=14,610; 4.41%)	8890/3: Leiomyosarcoma, NOS	6663	45.61
	8930/3: Endometrial stromal sarcoma, NOS	2076	14.21
	8933/3: Adenosarcoma	1703	11.66
	8931/3: Endometrial stromal sarcoma, low grade	1586	10.86
	8800/3: Sarcoma, NOS	777	5.32
	8891/3: Epithelioid leiomyosarcoma	414	2.83
	8935/3: Stromal sarcoma, NOS	321	2.20
	8896/3: Myxoid leiomyosarcoma	275	1.88
	8805/3: Undifferentiated sarcoma	210	1.44
	8900/3: Rhabdomyosarcoma, NOS	206	1.41
	8801/3: Spindle cell sarcoma	96	0.66
	8901/3: Pleomorphic rhabdomyosarcoma, adult type	59	0.40
	8802/3: Giant cell sarcoma	49	0.34
	8910/3: Embryonal rhabdomyosarcoma, NOS	37	0.25
	8804/3: Epithelioid sarcoma	34	0.23
	8902/3: Mixed type rhabdomyosarcoma	17	0.12
	9120/3: Hemangiosarcoma	15	0.10
	8803/3: Small cell sarcoma	8	0.05
	8920/3: Alveolar rhabdomyosarcoma	7	0.05
	9180/3: Osteosarcoma, NOS	7	0.05
	9260/3: Ewing sarcoma	7	0.05
	9220/3: Chondrosarcoma, NOS	6	0.04
	8810/3: Fibrosarcoma, NOS	4	0.03
	8850/3: Liposarcoma, NOS	4	0.03
	8936/3: Gastrointestinal stromal sarcoma	4	0.03
	8840/3: Myxosarcoma	3	0.02
	8895/3: Myosarcoma	3	0.02
	8912/3: Spindle cell rhabdomyosarcoma	3	0.02
	8855/3: Mixed liposarcoma	2	0.01
	8858/3: Dedifferentiated liposarcoma	2	0.01

	8811/3: Fibromyxosarcoma	1	0.01
	8814/3: Infantile fibrosarcoma	1	0.01
	8851/3: Liposarcoma, well differentiated	1	0.01
	8852/3: Myxoid liposarcoma	1	0.01
	8853/3: Round cell liposarcoma	1	0.01
	8854/3: Pleomorphic liposarcoma	1	0.01
	8860/3: Angiomyoliposarcoma	1	0.01
	8921/3: Rhabdomyosarcoma with ganglionic differentiation	1	0.01
	8991/3: Embryonal sarcoma	1	0.01
	9133/3: Epithelioid hemangioendothelioma, malignant	1	0.01
	9231/3: Myxoid chondrosarcoma	1	0.01
	9561/3: MPNST with rhabdomyoblastic differentiation	1	0.01
Other Uterine Cancers (n=7,543; 2.28%)	8010/3: Carcinoma, NOS	3038	35.17
	8000/3: Neoplasm, malignant	1122	12.99
	8070/3: Squamous cell carcinoma, NOS	1065	12.33
	8020/3: Carcinoma, undifferentiated, NOS	558	6.46
	8246/3: Neuroendocrine carcinoma, NOS	232	2.69
	8041/3: Small cell carcinoma, NOS	222	2.57
	9100/3: Choriocarcinoma, NOS	180	2.08
	8482/3: Mucinous adenocarcinoma, endocervical type	94	1.09
	8071/3: Squamous cell carcinoma, keratinizing, NOS	87	1.01
	8072/3: Squamous cell carcinoma, large cell, nonkeratinizing, NOS	72	0.83
	8013/3: Large cell neuroendocrine carcinoma	71	0.82
	8574/3: Adenocarcinoma with neuroendocrine differentiation	68	0.79
	9105/3: Trophoblastic tumor, epithelioid	48	0.56
	8033/3: Pseudosarcomatous carcinoma	46	0.53
	8046/3: Non-small cell carcinoma	45	0.52
	8032/3: Spindle cell carcinoma, NOS	40	0.46
	9110/3: Mesonephroma, malignant	35	0.41
	9473/3: Primitive neuroectodermal tumor	28	0.32
	8005/3: Malignant tumor, clear cell type	27	0.31
	8012/3: Large cell carcinoma, NOS	25	0.29

8384/3: Adenocarcinoma, endocervical type	22	0.25
8021/3: Carcinoma, anaplastic, NOS	21	0.24
8963/3: Malignant rhabdoid tumor	20	0.23
8031/3: Giant cell carcinoma	16	0.19
8120/3: Transitional cell carcinoma, NOS	16	0.19
8940/3: Mixed tumor, malignant, NOS	16	0.19
8004/3: Malignant tumor, spindle cell type	15	0.17
8045/3: Combined small cell carcinoma	15	0.17
8052/3: Papillary squamous cell carcinoma	15	0.17
8130/3: Papillary transitional cell carcinoma	14	0.16
9071/3: Yolk sac tumor	14	0.16
8015/3: Glassy cell carcinoma	12	0.14
8450/3: Papillary cystadenocarcinoma, NOS	12	0.14
8990/3: Mesenchymoma, malignant	12	0.14
9364/3: Peripheral neuroectodermal tumor	12	0.14
8022/3: Pleomorphic carcinoma	11	0.13
8230/3: Solid carcinoma, NOS	11	0.13
8083/3: Basaloid squamous cell carcinoma	9	0.10
8575/3: Metaplastic carcinoma, NOS	9	0.10
8830/3: Malignant fibrous histiocytoma	7	0.08
9014/3: Serous adenocarcinofibroma	7	0.08
9581/3: Alveolar soft part sarcoma	6	0.07
8051/3: Verrucous carcinoma, NOS	5	0.06
8074/3: Squamous cell carcinoma, spindle cell	5	0.06
8076/3: Squamous cell carcinoma, micro-invasive	5	0.06
8244/3: Mixed adenoneuroendocrine carcinoma (ICD-O-3 update)	5	0.06
8320/3: Granular cell carcinoma	5	0.06
8590/3: Sex cord-gonadal stromal tumor, malignant, NOS	5	0.06
8714/3: Perivascular epithelioid tumor, malignant	5	0.06
8098/3: Adenoid basal cell carcinoma	4	0.05
8240/3: Carcinoid tumor, NOS	4	0.05
8523/3: Infiltrating duct mixed with other types of carcinoma	4	0.05

8576/3: Hepatoid adenocarcinoma	4	0.05
9080/3: Teratoma, malignant, NOS	4	0.05
9104/3: Malignant placental site trophoblastic tumor	4	0.05
8001/3: Tumor cells, malignant	3	0.03
8073/3: Squamous cell carcinoma, small cell, nonkeratinizing	3	0.03
8143/3: Superficial spreading adenocarcinoma	3	0.03
8144/3: Adenocarcinoma, intestinal type	3	0.03
8313/3: Clear cell adenocarcinofibroma	3	0.03
8620/3: Granulosa cell tumor, malignant	3	0.03
8720/3: Malignant melanoma, NOS	3	0.03
8825/3: Myofibroblastoma, malignant	3	0.03
8897/3: Malignant tumor of smooth muscle	3	0.03
8934/3: Carcinofibroma	3	0.03
9085/3: Mixed germ cell tumor	3	0.03
9101/3: Choriocarcinoma combined with other germ cell elements	3	0.03
9150/3: Hemangiopericytoma, malignant	3	0.03
8014/3: Large cell carcinoma with rhabdoid phenotype	2	0.02
8030/3: Giant cell and spindle cell carcinoma	2	0.02
8082/3: Lymphoepithelial carcinoma	2	0.02
8123/3: Basaloid carcinoma	2	0.02
8201/3: Cribriform carcinoma, NOS	2	0.02
8290/3: Oxyphilic adenocarcinoma	2	0.02
8562/3: Epithelial-myoepithelial carcinoma	2	0.02
8580/3: Thymoma, malignant, NOS	2	0.02
8680/3: Paraganglioma, malignant	2	0.02
8960/3: Nephroblastoma, NOS	2	0.02
9102/3: Malignant teratoma, trophoblastic	2	0.02
8003/3: Malignant tumor, giant cell type	1	0.01
8011/3: Epithelioma, malignant	1	0.01
8040/3: Tumorlet, malignant	1	0.01
8084/3: Squamous cell carcinoma, clear cell type	1	0.01
8200/3: Adenoid cystic carcinoma	1	0.01

	8220/3: Adenocarcinoma in adenomatous polyposis coli	1	0.01
	8221/3: Adenocarcinoma in multiple adenomatous polyps	1	0.01
	8249/3: Atypical carcinoid tumor	1	0.01
	8280/3: Acidophil carcinoma	1	0.01
	8337/3: Insular carcinoma	1	0.01
	8343/3: Papillary carcinoma, encapsulated	1	0.01
	8360/3: Multiple endocrine adenomas, malignant	1	0.01
	8370/3: Adrenal cortical carcinoma	1	0.01
	8410/3: Sebaceous adenocarcinoma	1	0.01
	8462/3: Papillary serous cystadenocarcinoma	1	0.01
	8463/3: Serous surface papillary carcinoma	1	0.01
	8507/3: Ductal carcinoma, micropapillary	1	0.01
	8510/3: Medullary carcinoma, NOS	1	0.01
	8591/3: Sex cord-gonadal stromas tumor, malignant	1	0.01
	8640/3: Sertoli cell carcinoma	1	0.01
	8806/3: Desmoplastic small round cell tumor	1	0.01
	9015/3: Mucinous adenocarcinofibroma	1	0.01
	9060/3: Dysgerminoma	1	0.01
	9065/3: Germ cell tumor, nonseminomatous	1	0.01
	9130/3: Hemangioendothelioma, malignant	1	0.01
	9251/3: Malignant giant cell tumor of soft parts	1	0.01
	9380/3: Glioma, malignant	1	0.01
	9508/3: Atypical teratoid/rhabdoid tumor	1	0.01

Abbreviations: International Classification of Disease for Oncology, third edition (ICD-O-3), Not otherwise specified (NOS)

Notes: A total of n=392,574 newly diagnosed uterine cancer cases were reported in the SEER 22 (excluding IL, and MA) database for the diagnosis years between 2000 to 2018 in women aged ≥30 years, regardless of the availability of the survival data. The histologic subtype was categorized based on the ICD-O-3 histology/behavior code.

[a] Percentage of each major histologic subtype classification was calculated using all cases as the denominator.

[b] Percentage of individual ICD-O-3 histology/behavior code was calculated using case counts of major histologic subtype classification (endometrioid, non-endometrioid, sarcoma, and other uterine cancers).

[c] Note that there could be misclassification of N=34,491cases of Adenocarcinoma, NOS, especially those diagnosed during earlier time periods, with some belonging to the non-endometrioid subtype.

Table S2. Overall Five-Year Relative Survival Rates (Not Standardized for Age, 2000-2019) of Women Diagnosed with Uterine Cancer across Demographic, Clinical and Socioeconomic Characteristics, among U.S. Women Aged ≥30 Years, Diagnosed Between Years 2000 and 2018, SEER 22

Population Characteristics		Cases, N (%)	Deaths, N	Survival Rate (95% CI) [a]	p-value [b]
Overall, All Women		333,013	79,782	80.2 (80.1, 80.4)	-
Age Groups (years)					
	30-49	44,305 (13.38)	4,999	88.6 (88.3, 89.0)	Reference
	50-69	193,802 (58.55)	36,691	82.7 (82.5, 82.9)	<0.001
	70-79	62,863 (18.99)	21,100	73.6 (73.1, 74.0)	<0.001
	≥80	30,043 (9.08)	16,992	64.0 (63.0, 64.9)	<0.001
Race/Ethnicity					
	NH-White	229,273 (68.85)	51,882	83.0 (82.8, 83.2)	Reference
	NH-Black	34,200 (10.27)	14,096	60.8 (60.2, 61.4)	<0.001
	NH-Asian/PI	21,209 (6.37)	3,890	81.9 (81.3, 82.5)	0.0908
	NH-NA/AN	1,615 (0.48)	338	83.2 (80.7, 85.5)	0.0673
	Hispanic	44,716 (13.43)	9,576	79.5 (79.0, 79.9)	<0.001
Histology					
	Endometrioid	247,240 (74.69)	39,919	89.1 (88.9, 89.3)	Reference
	Non-Endometrioid	61,620 (18.62)	28,525	55.3 (54.9, 55.8)	<0.001
	Sarcoma	14,610 (4.41)	7,060	51.3 (50.4, 52.2)	<0.001
	Other	7,543 (2.28)	4,278	45.2 (44.0, 46.5)	<0.001
Stage at Diagnosis [c]					
	Localized	184,719 (69.68)	20,751	93.9 (93.7, 94.1)	Reference
	Regional	55,805 (21.05)	19,463	67.1 (66.7, 67.6)	<0.001
	Distant	24,583 (9.27)	19,475	17.5 (16.9, 18.0)	<0.001
%<HS Education [d]					
	Tertile 1 (<9.48%)	96,211 (29.07)	21,193	82.6 (82.3, 82.9)	Reference
	Tertile 2 (9.48-14.74%)	119,901 (36.23)	28,580	80.7 (80.4, 80.9)	<0.001
	Tertile 3 (>14.74%)	114,873 (34.70)	30,000	77.8 (77.5, 78.1)	<0.001
%<150 Percent Poverty [d]					
	Tertile 1 (20.92%)	149,375 (45.13)	33,111	82.2 (82.0, 82.5)	Reference
	Tertile 2 (20.92-28.12%)	131,906 (39.85)	32,518	79.6 (79.3, 79.9)	<0.001
	Tertile 3 (>28.12%)	49,704 (15.02)	14,144	75.9 (75.4, 76.3)	<0.001
%Unemployment [d]					

Tertile 1 (<4.07%)	45,981 (13.89)	10,014	82.9 (82.5, 83.4)	Reference
Tertile 2 (4.07%-5.86%)	166,282 (50.24)	39,305	80.8 (80.5, 81.0)	<0.001
Tertile 3 (>5.86%)	118,722 (35.87)	30,454	78.4 (78.1, 78.7)	<0.001
Median Household Income (USD) [d]				
Tertile 1 (<$47,060)	28,623 (8.65)	8,103	76.3 (75.7, 76.9)	Reference
Tertile 2 ($47,060-$56,590)	43,064 (13.01)	10,620	80.6 (80.2, 81.1)	<0.001
Tertile 3 (>$56,590)	259,298 (78.34)	61,050	80.6 (80.4, 80.8)	<0.001
% Urban [d]				
Tertile 1 (<24.53%)	9,019 (2.72)	2,237	80.6 (79.5, 81.6)	Reference
Tertile 2 (24.53-57.02%)	30,149 (9.11)	7,302	81.3 (80.7, 81.8)	0.93385
Tertile 3 (>57.02%)	291,819 (88.17)	70,234	80.1 (79.9, 80.3)	0.11071

Notes. Percentages were calculated using non-missing data.

Abbreviations: CI, Confidence Interval; NA/AN, Native American/ Alaska Native; NH, Non-Hispanic; PI, Pacific Islander, SES, Socioeconomic status

[a] We included first primary uterine cancer cases diagnosed between 2000 and 2018, with survival assessed based on all-cause mortality estimated through 2019.

[b] P-values (Two tailed) indicates the statistical difference between two 5-year relative survival rates computed from the z-statistic from the SEER*Stat program, with the formula $p=2\times(1-\Phi(|z|))$, where $\Phi(x)$= standard normal distribution's cumulative distribution function, and $|z|$ = absolute z-score.

[c] Stage was not available prior to 2004, resulting in a total of n=54,918 missing data points. Additionally, we set n=10,978 (unknown/not-staged), and n=10 (in-situ) tumors diagnosed from 2004 to 2018 as missing.

[d] All county-level socioeconomic characteristics, except % urban (2010 U.S. Census County Attributes), were sourced from the ACS database (2015-2019) linked to SEER 22. Selected variables ranged as follows: %<HS Education (1.12% to 73.56%), %<150 Poverty (4.84% to 68.30%), % Unemployed (0% to 27.15%), Median Household Income ($21,500 to $142,300), and % urban (0 to 100% urban). For any given SES characteristic, except for the median household income, tertile 1 corresponds to the first 33% of counties with the highest SES, while tertiles 2 and 3 correspond to medium and the lowest SES. Median household income is inverse. Tertile distribution corresponds to the county-level SES characteristic and not the distribution of case numbers.

Table S3. Race/Ethnicity Specific Age-Standardized Five-Year Relative Survival Rates (2000-2019) of Women Diagnosed with Uterine Cancer across Socioeconomic Characteristics, Overall and by Major Histologic Subtypes (Endometrioid and Non-Endometrioid Cancer) among U.S. Women Aged ≥30 Years, Diagnosed between Years 2000 and 2018, SEER 22

**SES** [a]	**Histology** [b]	**Age-Standardized 5-Yr Relative Survival Rate (95% CI)** [c]					
		**All Racial/ Ethnic Groups**	**NH-White**	**NH-Black**	**NH-Asian/PI**	**NH-NA/AN**	**Hispanic**
**%<HS Education**							
Tertile 1	Overall (All Subtypes)	80.2	81.8	61.0	76.0	80.1	74.4
		(79.8, 80.6)	(81.4, 82.2)	(59.1, 62.8)	(74.1, 77.8)	(72.8, 85.6)	(71.8, 76.8)
Tertile 2	Overall (All Subtypes)	78.3***	81.6**	57.5**	76.2^{ns}	78.9^{ns}	74.2^{ns}
		(78.0, 78.7)	(81.2, 82.0)	(56.4, 58.7)	(74.6, 77.8)	(70.8, 84.9)	(72.9, 75.5)
Tertile 3	Overall (All Subtypes)	74.7***	79.7***	56.7***	75.0^{ns}	71.0^{ns}	72.5***
		(74.3, 75.1)	(79.3, 80.2)	(55.7, 57.7)	(73.3, 76.7)	(63.2, 77.5)	(71.6, 73.4)
Tertile 1	Endometrioid	88.5	89.3	76.3	86.0	87.7	83.8
		88.1, 89.0)	(88.8, 89.7)	(73.5, 78.8)	(83.7, 88.0)	(80.4, 92.5)	(80.6, 86.5)
Tertile 2	Endometrioid	87.6***	89.3^{ns}	72.1**	86.4^{ns}	86.8^{ns}	84.2^{ns}
		(87.2, 88.0)	(88.8, 89.7)	(70.4, 73.8)	(84.4, 88.1)	(75.7, 93.1)	(82.6, 85.7)
Tertile 3	Endometrioid	85.2***	87.8***	72.8***	84.8^{ns}	79.4^{ns}	82.7*
		(84.8, 85.7)	(87.2, 88.3)	(71.3, 74.2)	(82.7, 86.7)	(69.4, 86.5)	(81.5, 83.7)
Tertile 1	Non-Endometrioid	58.1	60.3	43.5	54.9	53.4	53.9^{ns}
		(57.2, 59.0)	(59.2, 61.3)	(40.3, 46.6)	(50.7, 58.9)	(39.4, 65.6)	(48.6, 58.8)
Tertile 2	Non-Endometrioid	57.3^{ns}	61.0^{ns}	43.1^{ns}	58.0^{ns}	58.3^{ns}	56.8^{ns}
		(56.5, 58.1)	(59.9, 61.9)	(41.1, 45.0)	(54.6, 61.2)	(44.6, 69.8)	(54.2, 59.2)
Tertile 3	Non-Endometrioid	52.5***	58.0**	40.7*	54.1^{ns}	50.6^{ns}	52.8
		(51.7, 53.3)	(56.8, 59.2)	(39.0, 42.5)	(50.6, 57.4)	(36.3, 63.3)	(51.0, 54.5)
**%<150 Percent Poverty**							
Tertile 1	Overall	79.8	81.8	59.9	76.7	81.4	74.8
		(79.5, 80.2)	(81.4, 82.1)	(58.5, 61.4)	(75.3, 78.0)	(73.9, 86.9)	(73.4, 76.1)
Tertile 2	Overall	77.0***	80.9***	58.7*	74.9**	77.2^{ns}	73.1**
		(76.6, 77.3)	(80.5, 81.3)	(57.6, 59.8)	(73.3, 76.5)	(68.8, 83.6)	(72.1, 74.1)
Tertile 3	Overall	72.9***	79.6***	54.1***	71.2^{ns}	67.6*	71.3***
		(72.3, 73.5)	(78.8, 80.3)	(52.8, 55.4)	(65.8, 75.9)	(59.2, 74.7)	(69.8, 72.7)
Tertile 1	Endometrioid	88.5	89.4	75.2	86.7	89.4	84.7

		(88.2, 88.9)	(89.0, 89.8)	(73.1, 77.2)	(85.1, 88.1)	(84.0, 93.1)	(82.9, 86.2)
Tertile 2	Endometrioid	86.7***	88.6***	74.4^{ns}	85.3^{ns}	86.5^{ns}	83.5**
		(86.3, 87.1)	(88.1, 89.0)	(72.8, 75.8)	(83.3, 87.1)	(74.2, 93.2)	(82.3, 84.7)
Tertile 3	Endometrioid	83.7***	87.5***	69.2***	76.0^{ns}	74.8^{ns}	80.7***
		(83.0, 84.4)	(86.7, 88.2)	(67.3, 71.0)	(69.1, 81.6)	(64.3, 82.6)	(78.9, 82.4)
Tertile 1	Non-Endometrioid	58.4	60.7	44.8	57.2	59.9	56.0
		(57.7, 59.1)	(59.8, 61.5)	(42.3, 47.2)	(54.3, 59.9)	(46.5, 70.9)	(53.2, 58.7)
Tertile 2	Non-Endometrioid	54.9***	59.7**	42.3^{ns}	53.6^{ns}	NC^{d}	53.4^{ns}
		(54.1, 55.6)	(58.7, 60.7)	(40.5, 44.1)	(50.4, 56.7)		(51.5, 55.3)
Tertile 3	Non-Endometrioid	50.8***	57.4**	39.0***	62.2^{ns}	50.2^{ns}	53.3**
		(49.6, 52.1)	(55.6, 59.1)	(36.8, 41.3)	(51.7, 71.1)	(31.8, 66.2)	(50.4, 56.2)
**%Unemployment**							
Tertile 1	Overall	80.5	82.1	59.9	76.7	NR^{d}	73.4
		(79.9, 81.1)	(81.5, 82.6)	(56.7, 63.0)	(74.4, 78.8)		(70.5, 76.1)
Tertile 2	Overall	78.3***	81.3*	58.5^{ns}	76.3^{ns}	81.2	73.8*
		(78.0, 78.6)	(80.9, 81.6)	(57.5, 59.6)	(74.7, 77.7)	(72.4, 87.4)	(72.7, 74.8)
Tertile 3	Overall	75.7***	80.5***	56.6*	74.6^{ns}	74.4	72.5***
		(75.3, 76.0)	(80.1, 81.0)	(55.6, 57.6)	(72.9, 76.2)	(68.6, 79.3)	(71.5, 73.5)
Tertile 1	Endometrioid	88.7	89.5	74.9	86.3	NC/NR^{d}	82.1
		(88.0, 89.3)	(88.8, 90.1)	(69.7, 79.3)	(83.7, 88.6)		(78.4, 85.2)
Tertile 2	Endometrioid	87.5***	88.9^{ns}	74.8^{ns}	86.0^{ns}	91.2	83.6^{ns}
		(87.1, 87.8)	(88.5, 89.2)	(73.3, 76.3)	(84.1, 87.6)	(76.9, 96.8)	(82.3, 84.8)
Tertile 3	Endometrioid	85.9***	88.5**	71.2**	85.1^{ns}	83.2	83.0^{ns}
		(85.4, 86.3)	(88.0, 89)	(69.7, 72.6)	(83.0, 87.0)	(75.7, 88.5)	(81.7, 84.1)
Tertile 1	Non-Endometrioid	59.1	60.9	44.0	56.2	NC/NR^{d}	57.7
		(57.7, 60.4)	(59.3, 62.4)	(37.9, 49.9)	(51.2, 60.9)		(51.9, 63.2)
Tertile 2	Non-Endometrioid	56.5**	60.3^{ns}	41.1^{ns}	57.7^{ns}	56.9	54.8^{ns}
		(55.8, 57.2)	(59.5, 61.2)	(39.3, 42.9)	(54.6, 60.7)	(43.6, 68.2)	(52.7, 56.9)
Tertile 3	Non-Endometrioid	53.7***	58.7^{ns}	42.4^{ns}	53.2^{ns}	53.7	52.9*
		(52.8, 54.4)	(57.6, 59.8)	(40.7, 44.1)	(49.9, 56.5)	(43.4, 62.9)	(50.9, 54.8)
**Median Household Income (USD)**							

Tertile 1	Overall	73.3	80.0	54.1	77.8	65.0	71.9
		(72.5, 74.1)	(79.0, 80.9)	(52.4, 55.8)	(66.0, 86.0)	(52.6, 75.0)	(70.1, 73.6)
Tertile 2	Overall	78.3***	80.8^{ns}	57.5***	62.6*	76.2^{ns}	72.0**
		(77.7, 78.9)	(80.1, 81.4)	(55.1, 59.8)	(55.6, 68.8)	(67.1, 83.2)	(69.7, 74.2)
Tertile 3	Overall	78.1***	81.4^{ns}	58.5***	76.1^{ns}	79.4*	73.4***
		(77.8, 78.3)	(81.1, 81.6)	(57.6, 59.3)	(75.1, 77.1)	(73.5, 84.2)	(72.6, 74.2)
Tertile 1	Endometrioid	83.8	87.7	68.6	80.7	74.9	80.7
		(82.8, 84.6)	(86.6, 88.7)	(66.1, 70.9)	(65.4, 89.8)	(58.0, 85.8)	(78.4, 82.8)
Tertile 2	Endometrioid	86.5***	87.8^{ns}	72.4**	67.0*	82.8^{ns}	81.9*
		(85.8, 87.2)	(87.1, 88.6)	(68.9, 75.7)	(58.1, 74.5)	(70.5, 90.3)	(79.1, 84.5)
Tertile 3	Endometrioid	87.6***	89.2*	74.2***	86.3^{ns}	89.2^{ns}	83.8***
		(87.3, 87.8)	(88.8, 89.5)	(73.0, 75.4)	(85.1, 87.4)	(80.9, 94.0)	(82.8, 84.7)
Tertile 1	Non-Endometrioid	50.5	56.4	38.4	NR^{d}	NC^{d}	54.6
		(48.8, 52.1)	(53.9, 58.7)	(35.2, 41.5)			(50.9, 58.1)
Tertile 2	Non-Endometrioid	56.8***	59.8^{ns}	42.7*	47.6	60.2	51.9^{ns}
		(55.4, 58.2)	(58.2, 61.4)	(38.7, 46.6)	(34.8, 59.3)	(44.1, 73)	(47, 56.6)
Tertile 3	Non-Endometrioid	56.2***	60.2^{ns}	42.7**	55.9	57.9	54.1^{ns}
		(55.6, 56.7)	(59.5, 60.9)	(41.3, 44.1)	(53.8, 58)	(47.6, 66.9)	(52.5, 55.7)
**% Urban**							
Tertile 1	Overall	77.8	79.6	56.5	NR^{d}	NC/NR^{d}	74.5
		(76.4, 79.1)	(78.1, 81.0)	(51.4, 61.3)			(64.7, 81.9)
Tertile 2	Overall	79.0^{ns}	81.0^{ns}	55.1^{ns}	75.7^{d}	71.4	73.6^{ns}
		(78.3, 79.7)	(80.3, 81.8)	(52.3, 57.8)	(60.8, 85.6)	(61.7, 79.0)	(69.0, 77.6)
Tertile 3	Overall	77.5^{ns}	81.2^{ns}	57.9^{ns}	75.8	78.2	73.1^{ns}
		(77.3, 77.8)	(81.0, 81.5)	(57.2, 58.6)	(74.8, 76.8)	(72.9, 82.5)	(72.4, 73.8)
Tertile 1	Endometrioid	86.0	87.1	68.0	NR^{d}	NC/NR^{d}	80.7
		(84.4, 87.5)	(85.3, 88.6)	(60.9, 74.2)			(68.9, 88.4)
Tertile 2	Endometrioid	87.1^{ns}	88.3^{ns}	68.0^{ns}	NR^{d}	78.8	82.9^{ns}
		(86.3, 87.8)	(87.4, 89.0)	(64.0, 71.6)		(66.4, 87.1)	(77.0, 87.4)
Tertile 3	Endometrioid	87.1^{ns}	89.0^{ns}	73.5^{ns}	85.8	87.5	83.2^{ns}
		(86.9, 87.4)	(88.7, 89.3)	(72.5, 74.6)	(84.6, 87)	(80.5, 92.1)	(82.3, 84)
Tertile 1	Non-Endometrioid	55.3	57.1	48.1	NC/NR^{d}	NC/NR^{d}	NR^{d}
		(52.1, 58.4)	(53.6, 60.5)	(39.8, 55.9)			
Tertile 2	Non-Endometrioid	57.2^{ns}	59.7^{ns}	38.8^{ns}	NR^{d}	58.1	55.7

		(55.5, 58.8)	(57.9, 61.5)	(33.3, 44.3)		(39.0, 73.1)	(46.3, 64.0)
Tertile 3	Non-Endometrioid	55.6 [ns]	60.0 [ns]	42.2 [ns]	55.8	56.5	54.1
		(55.1, 56.1)	(59.3, 60.7)	(40.9, 43.4)	(53.7, 57.8)	(47.1, 64.9)	(52.7, 55.5)

Notes. Percentages were calculated using non-missing data.

Abbreviations: CI, Confidence Interval; NA/AN, Native American/ Alaska Native; NH, Non-Hispanic; PI, Pacific Islander.

[a] All county-level socioeconomic characteristics, except % urban (2010 U.S. Census County Attributes), were sourced from the ACS database (2015-2019) linked to SEER 22. SES variable tertile cut-offs with the range: %<HS Education (1.12% to 73.56%; T1 <9.48%, T2 9.48-14.74%, and T3 >14.74%), %<150 Poverty (4.84% to 68.30%; T1 <20.92%, T2 20.92-28.12%, and T3 >28.12%), % Unemployed (0% to 27.15%; T1 <4.07%, T2 4.07-5.86%, T3 >5.86%), Median Household Income ($21,500 to $142,300; T1 <$47,060, T2 $47,060-$56,590, T3 >$56,590), and % urban (0 to 100% urban; <24.53%, T2 24.53-57.02%, T3 >57.02%). For any given SES characteristic, except for the median household income, tertile 1 corresponds to the first 33% of counties with the highest SES, while tertiles 2 and 3 correspond to medium and the lowest SES. Median household income is inverse. Tertile distribution corresponds to the county-level SES characteristic and not the distribution of case numbers.

[c] P-values (Two tailed) indicates the statistical difference between two 5-year relative survival rates computed from the z-statistic from the SEER*Stat program, with the formula $p=2\times(1-\Phi(|z|))$, where $\Phi(x)$= standard normal distribution's cumulative distribution function, and $|z|$ = absolute z-score. P-value symbols: ns (not significant), * (<0.05), ** (<0.01), and *** (<0.001); Tertile 1 (reference).

[d] Not Computed (NC) indicates that the statistic could not be computed in SEER*Stat and Not Reported (NR) when survival estimates not shown due to having fewer than 16 cases/ deaths [26].

Table S4. Number of Women Diagnosed with Uterine Cancer and Deaths across Race/Ethnicity, Socioeconomic Characteristics and Histology, among U.S. Women Aged ≥30 Years, Diagnosed between Years 2000 and 2018, SEER 22

SES [a]	Histology	Sample Size	Racial and Ethnic Groups				
			NH-White	NH-Black	NH-Asian/PI	NH-NA/AN	Hispanic
**%<HS Education**							
Tertile 1	Overall	N cases (%)	80,797 (35.24)	5,239 (15.32)	5,819 (27.44)	498 (30.84)	3,858 (8.63)
		N deaths	17,290	1,979	1,090	94	740
Tertile 2	Overall	N cases (%)	86,252 (37.62)	12,586 (36.80)	8,153 (38.44)	533 (33.00)	12,377 (27.68)
		N deaths	19,312	5,185	1,442	111	2,530
Tertile 3	Overall	N cases (%)	62,204 (27.13)	16,375 (47.88)	7,235 (34.12)	584 (36.16)	28,475 (63.69)
		N deaths	15,274	6,932	1,357	133	6,304
Tertile 1	Endometrioid	N cases (%)	63,638 (35.61)	2,852 (15.58)	4,498 (28.07)	391 (30.96)	2,913 (8.85)
		N deaths	9,577	662	512	51	350
Tertile 2	Endometrioid	N cases (%)	66,961 (37.47)	6,884 (37.62)	6,123 (38.21)	415 (32.86)	8,970 (27.24)
		N deaths	10,600	1,878	630	61	1,088
Tertile 3	Endometrioid	N cases (%)	48,102 (26.92)	8,565 (46.80)	5,404 (33.72)	457 (36.18)	21,048 (63.92)
		N deaths	8,535	2,338	615	74	2,943
Tertile 1	Non-Endometrioid	N cases (%)	12,973 (34.19)	1,756 (14.69)	905 (24.59)	71 (28.63)	641 (8.22)
		N deaths	5,635	966	376	31	260
Tertile 2	Non-Endometrioid	N cases (%)	14,525 (38.28)	4,297 (35.96)	1,447 (39.31)	84 (33.87)	2,318 (29.74)
		N deaths	6,323	2,437	535	31	931
Tertile 3	Non-Endometrioid	N cases (%)	10,447 (27.53)	5,897 (49.35)	1,329 (36.10)	93 (37.50)	4,836 (62.04)
		N deaths	4,849	3,419	534	41	2,156
**%<150 Percent Poverty**							
Tertile 1	Overall	N cases (%)	116,264 (50.71)	8,493 (24.83)	12,073 (56.93)	642 (39.75)	11,903 (26.62)
		N deaths	25,219	3,288	2,165	124	2,315
Tertile 2	Overall	N cases (%)	84,374 (36.80)	15,856 (46.36)	8,121 (38.29)	530 (32.82)	23,025 (51.50)
		N deaths	19,576	6,452	1,535	102	4,853
Tertile 3	Overall	N cases (%)	28,615 (12.48)	9,851 (28.80)	1,013 (4.78)	443 (27.43)	9,782 (21.88)
		N deaths	7,081	4,356	189	112	2,406
Tertile 1	Endometrioid	N cases (%)	90,655 (50.73)	4,539 (24.80)	9,192 (57.36)	505 (39.98)	8,782 (26.67)

		N deaths	13,703	1,100	983	70	998
Tertile 2	Endometrioid	N cases (%)	65,674 (36.75)	8,613 (47.06)	6,063 (37.83)	407 (32.22)	16,942 (51.45)
		N deaths	10,935	2,249	680	50	2,194
Tertile 3	Endometrioid	N cases (%)	22,372 (12.52)	5,149 (28.14)	770 (4.80)	351 (27.79)	7,207 (21.89)
		N deaths	4,074	1,529	94	66	1,189
Tertile 1	Non-Endometrioid	N cases (%)	19,371 (51.05)	2,957 (24.74)	2,021 (54.90)	91 (36.69)	2,100 (26.94)
		N deaths	8,369	1,610	775	34	845
Tertile 2	Non-Endometrioid	N cases (%)	13,977 (36.83)	5,443 (45.55)	1,497 (40.67)	91 (36.69)	4,055 (52.02)
		N deaths	6,286	3,111	610	38	1,742
Tertile 3	Non-Endometrioid	N cases (%)	4,597 (12.11)	3,550 (29.71)	163 (4.43)	66 (26.61)	1,640 (21.04)
		N deaths	2,152	2,101	60	31	760
**%Unemploy-ment**							
Tertile 1	Overall	N cases (%)	36,764 (16.04)	1,699 (4.97)	4,280 (20.18)	94 (5.82)	3,144 (7.03)
		N deaths	7,925	655	812	18	604
Tertile 2	Overall	N cases (%)	121,227 (52.88)	15,608 (45.64)	9,335 (44.02)	580 (35.91)	19,532 (43.69)
		N deaths	27,134	6,348	1,607	110	4,106
Tertile 3	Overall	N cases (%)	71,262 (31.08)	16,893 (49.39)	7,592 (35.80)	941 (58.27)	22,034 (49.28)
		N deaths	16,817	7,093	1,470	210	4,864
Tertile 1	Endometrioid	N cases (%)	28,786 (16.11)	887 (4.85)	3,339 (20.84)	75 (5.94)	2,348 (7.13)
		N deaths	4,371	214	394	NR [b]	285
Tertile 2	Endometrioid	N cases (%)	94,511 (52.89)	8,410 (45.95)	7,016 (43.78)	455 (36.03)	14,367 (43.63)
		N deaths	15,045	2,155	718	58	1,876
Tertile 3	Endometrioid	N cases (%)	55,404 (31.00)	9,004 (49.20)	5,670 (35.38)	733 (58.04)	16,216 (49.24)
		N deaths	9,296	2,509	645	116	2,220
Tertile 1	Non-Endometrioid	N cases (%)	5,989 (15.78)	601 (5.03)	648 (17.60)	NR [b]	528 (6.77)
		N deaths	2,603	329	267	NR [b]	204
Tertile 2	Non-Endometrioid	N cases (%)	19,985 (52.67)	5,368 (44.92)	1,629 (44.25)	80 (32.26)	3,403 (43.66)
		N deaths	8,757	3,085	599	31	1447
Tertile 3	Non-Endometrioid	N cases (%)	11,971 (31.55)	5,981 (50.05)	1,404 (38.14)	155 (62.50)	3,864 (49.57)
		N deaths	5,447	3,408	579	68	1696

**Median Household Income (USD)**							
Tertile 1	Overall	N cases (%)	16,529 (7.21)	5,377 (15.72)	219 (1.03)	248 (15.36)	6,250 (13.98)
		N deaths	4,011	2,418	40	61	1,573
Tertile 2	Overall	N cases (%)	34,541 (15.07)	3,302 (9.65)	542 (2.56)	381 (23.59)	4,298 (9.61)
		N deaths	8,104	1,347	137	80	952
Tertile 3	Overall	N cases (%)	178,183 (77.72)	25,521 (74.62)	20,446 (96.41)	986 (61.05)	34,162 (76.41)
		N deaths	39,761	10,331	3,712	197	7,049
Tertile 1	Endometrioid	N cases (%)	13,138 (7.35)	2,887 (15.78)	163 (1.02)	191 (15.12)	4,594 (13.95)
		N deaths	2,369	893	19	34	794
Tertile 2	Endometrioid	N cases (%)	27,338 (15.30)	1,848 (10.10)	406 (2.53)	302 (23.91)	3,294 (10.00)
		N deaths	4,778	512	73	46	483
Tertile 3	Endometrioid	N cases (%)	138,225 (77.35)	13,566 (74.13)	15,456 (96.45)	770 (60.97)	25,043 (76.05)
		N deaths	21,565	3,473	1,665	106	3,104
Tertile 1	Non-Endometrioid	N cases (%)	2,492 (6.57)	1,900 (15.90)	38 (1.03)	42 (16.94)	1,045 (13.41)
		N deaths	1,173	1,151	NR [b]	20	481
Tertile 2	Non-Endometrioid	N cases (%)	5,365 (14.14)	1,037 (8.68)	104 (2.83)	57 (22.98)	615 (7.89)
		N deaths	2,412	585	49	23	272
Tertile 3	Non-Endometrioid	N cases (%)	30,088 (79.29)	9,013 (75.42)	3,539 (96.14)	149 (60.08)	6,135 (78.70)
		N deaths	13,222	5,086	1,386	60	2,594
**% Urban**							
Tertile 1	Overall	N cases (%)	8,107 (3.54)	630 (1.84)	25 (0.12)	24 (1.49)	231 (0.52)
		N deaths	1,897	273	NR [b]	NR [b]	54
Tertile 2	Overall	N cases (%)	26,450 (11.54)	2,010 (5.88)	135 (0.64)	320 (19.81)	1,234 (2.76)
		N deaths	6,038	912	18	70	264
Tertile 3	Overall	N cases (%)	194,696 (84.93)	31,560 (92.28)	21,047 (99.25)	1,271 (78.70)	43,245 (96.72)
		N deaths	43,941	12,911	3,863	263	9,256
Tertile 1	Endometrioid	N cases (%)	6,424 (3.59)	362 (1.98)	19 (0.12)	21 (1.66)	175 (0.53)
		N deaths	1,095	113	NR [b]	NR [b]	30
Tertile 2	Endometrioid	N cases (%)	20,860 (11.67)	1,119 (6.11)	100 (0.62)	249 (19.71)	926 (2.81)
		N deaths	3,500	369	NR [b]	41	126
Tertile 3	Endometrioid	N cases (%)	151,417 (84.73)	16,820 (91.91)	15,906 (99.26)	993 (78.62)	31,830 (96.66)
		N deaths	24,117	4,396	1,745	142	4,225

Tertile 1	Non-Endometrioid	N cases (%)	1,247 (3.29)	212 (1.77)	NR [b]	NR [b]	35 (0.45)
		N deaths	575	118	NR [b]	NR [b]	NR [b]
Tertile 2	Non-Endometrioid	N cases (%)	4,208 (11.09)	656 (5.49)	24 (0.65)	52 (20.97)	199 (2.55)
		N deaths	1,868	399	NR [b]	21	82
Tertile 3	Non-Endometrioid	N cases (%)	32,490 (85.62)	11,082 (92.74)	3,651 (99.19)	194 (78.23)	7,561 (97.00)
		N deaths	14,364	6,305	1436	80	3250

Notes. Percentages were calculated using non-missing data.

Abbreviations: NA/AN, Native American/ Alaska Native; NH, Non-Hispanic; PI, Pacific Islander.

[a] All county-level socioeconomic characteristics, except % urban (2010 U.S. Census County Attributes), were sourced from the ACS database (2015-2019) linked to SEER 22. SES variable tertile cut-offs with the range: %<HS Education (1.12% to 73.56%; T1 <9.48%, T2 9.48-14.74%, and T3 >14.74%), %<150 Poverty (4.84% to 68.30%; T1 <20.92%, T2 20.92-28.12%, and T3 >28.12%), % Unemployed (0% to 27.15%; T1 <4.07%, T2 4.07-5.86%, T3 >5.86%), Median Household Income ($21,500 to $142,300; T1 <$47,060, T2 $47,060-$56,590, T3 >$56,590), and % urban (0 to 100% urban; <24.53%, T2 24.53-57.02%, T3 >57.02%). For any given SES characteristic, except for the median household income, tertile 1 corresponds to the first 33% of counties with the highest SES, while tertiles 2 and 3 correspond to medium and the lowest SES. Median household income is inverse. Tertile distribution corresponds to the county-level SES characteristic and not the distribution of case numbers.

[b] Not Reported (NR) indicates fewer than 16 cases or fewer than 16 deaths [26].

Table S5. Age-Standardized Five-Year Relative Survival Rates (2000-2019) of Women Diagnosed with Uterine Cancer by U.S. Geographic Region and Socioeconomic Characteristics among U.S. Women Aged ≥30 Years, Diagnosed between Years 2000 and 2018, SEER 22

SES and U.S. Geographic Region		N cases (N deaths)	5-Yr Relative Survival Rate (95% CI)
**U.S. Geographic Region** [a]			
	Northeast	110,757 (27754)	76.9 (76.5, 77.2)
	Midwest (only Iowa)	10,365 (2371)	80.8 (79.7, 82.0)
	South	89,734 (22476)	76.6 (76.1, 77.1)
	West	120,157 (27181)	79.1 (78.7, 79.4)
**Region and %<HS Education** [a,b]			
	**Northeast**		
	Tertile 1	44,455 (10,100)	79.6 (79.1, 80.2)
	Tertile 2**	43,088 (10,544)	78.0 (77.4, 78.6)
	Tertile 3**	23,204 (7,106)	69.4 (68.6, 70.2)
	**South**		
	Tertile 1	15,481 (3,500)	78.9 (77.7, 80.0)
	Tertile 2**	30,627 (7,681)	77.0 (76.2, 77.8)
	Tertile 3**	43,625 (11,295)	75.5 (74.8, 76.1)
	**West and Midwest**		
	Tertile 1	36,275 (7,593)	81.7 (81.0, 82.3)
	Tertile 2**	46,186 (10,355)	79.6 (79.0, 80.2)
	Tertile 3**	48,044 (11,599)	76.9 (76.3, 77.5)
**Region and %<150 percent Poverty** [a,b]			
	**Northeast**		
	Tertile 1	61,403 (14,297)	79.0 (78.5, 79.5)
	Tertile 2**	35,490 (8,986)	76.6 (76.0, 77.2)
	Tertile 3**	13,854 (4,467)	67.8 (66.7, 68.8)
	**South**		
	Tertile 1	20,259 (4,286)	80.1 (79.0, 81.0)
	Tertile 2**	43,102 (10,863)	76.7 (76.0, 77.3)
	Tertile 3**	26,372 (7,327)	74.0 (73.2, 74.8)
	**West and Midwest**		
	Tertile 1	67,713 (14,528)	80.7 (80.2, 81.1)
	Tertile 2**	53,314 (12,669)	77.6 (77.0, 78.1)

Tertile 3**	9,478 (2,350)	77.8 (76.5, 79.1)
**Region and %Unemployment [a,b]**		
**Northeast**		
Tertile 1	11,908 (2,696)	79.9 (78.8, 80.9)
Tertile 2**	66,496 (16,203)	77.6 (77.1, 78.0)
Tertile 3**	32,343 (8,851)	74.2 (73.5, 74.9)
**South**		
Tertile 1	13,346 (2,787)	81.4 (80.2, 82.5)
Tertile 2**	51,071 (12,597)	77.0 (76.4, 77.6)
Tertile 3**	25,316 (7,092)	73.4 (72.5, 74.2)
**West and Midwest**		
Tertile 1	20,727 (4,531)	80.4 (79.5, 81.2)
Tertile 2	48,715 (10,505)	80.9 (80.3, 81.4)
Tertile 3**	61,063 (14,511)	77.5 (76.9, 78.0)
**Region and Median Household Income [a,b]**		
**Northeast**		
Tertile 1	4,573 (1,446)	68.0 (66.1, 69.8)
Tertile 2**	8,088 (1,806)	80.8 (79.4, 82.1)
Tertile 3**	98,086 (24,498)	76.9 (76.6, 77.3)
**South**		
Tertile 1	20,939 (5,876)	73.7 (72.8, 74.6)
Tertile 2**	20,402 (5,305)	76.4 (75.5, 77.3)
Tertile 3**	48,392 (11,295)	78.0 (77.4, 78.6)
**West and Midwest**		
Tertile 1	3,111 (781)	78.1 (75.8, 80.2)
Tertile 2	14,574 (3,509)	79.3 (78.3, 80.3)
Tertile 3*	112,820 (25,257)	79.2 (78.9, 79.6)

[a] SEER registries were classified into geographic regions including Northeast (Connecticut, New Jersey, New York), Midwest (Iowa), South (Georgia, Kentucky, Louisiana and Texas), and West (Alaska, California, Hawaii, Idaho, New Mexico, Utah, Washington). Later combined West and Midwest together in the stratified analysis.

[b] All county-level socioeconomic characteristics were sourced from the ACS database (2015-2019) linked to SEER 22. SES variable tertile cut-offs with the range: %<HS Education (1.12% to 73.56%; T1 <9.48%, T2 9.48-14.74%, and T3 >14.74%), %<150 Poverty (4.84% to 68.30%; T1 <20.92%, T2 20.92-28.12%, and T3 >28.12%), % Unemployed (0% to 27.15%; T1 <4.07%, T2 4.07-5.86%, T3 >5.86%), and Median Household Income ($21,500 to $142,300; T1 <$47,060, T2 $47,060-$56,590, T3 >$56,590). For any given SES characteristic, except for the median household income, tertile 1 corresponds to the

first 33% of counties with the highest SES, while tertiles 2 and 3 correspond to medium and the lowest SES. Median household income is inverse. Tertile distribution corresponds to the county-level SES characteristic and not the distribution of case numbers.

[c] P-values (Two tailed) indicates the statistical difference between two 5-year relative survival rates computed from the z-statistic from the SEER*Stat program, with the formula $p=2\times(1-\Phi(|z|))$, where $\Phi(x)$= standard normal distribution's cumulative distribution function, and $|z|$ = absolute z-score. P-value symbols: ** (<0.001), * (≤0.01); Tertile 1 (reference).
